# TGF-β inhibitor SB431542 suppresses SARS-CoV-2 replication through multistep inhibition

**DOI:** 10.1128/jvi.00529-25

**Published:** 2025-08-29

**Authors:** Assim Verma, Himanshu Kamboj, Garvit Kumar, Nitin Khandelwal, Benjamin E. Mayer, Jitender Rathee, Yogesh Chander, Alka Nokhwal, Shweta Dhanda, Ram Kumar, Ramesh Kumar Dedar, Sandeep Kumar Bejjanki, Deepti Parashar, Gayathri Pananghat, Bhupendra Nath Tripathi, Riyesh Thachamvally, Shalini Sharma, Naveen Kumar

**Affiliations:** 1 National Centre for Veterinary Type Cultures, ICAR-National Research Centre on Equines, Hisar, India; 2 ICMR-National Institute of Virology 29620, Pune, India; 3 Interuniversity Microelectronics Centre, Leuven, Belgium; 4 Department of Biology, Indian Institute of Science Education and Research 193158, Pune, India; 5 Science and Engineering Research Board (SERB), Teachers Associateship for Research Excellence (TARE) fellow at National Centre for Veterinary Type Cultures, Hisar, India; Loyola University Chicago - Health Sciences Campus, Maywood, Illinois, USA

**Keywords:** SARS-CoV-2, antiviral

## Abstract

The coronavirus disease 2019 (COVID-19) pandemic highlighted the critical need for broad-spectrum antivirals with high resistance barriers. Here, we demonstrate that SB431542, a selective TGF-β receptor I (ALK5) inhibitor, exhibits potent antiviral activity against severe acute respiratory syndrome coronavirus 2 (SARS-CoV-2) through unprecedented multitargeted mechanisms. Through comprehensive *in vitro*, isothermal titration calorimetry, and *in silico* analyses, we identified that SB431542 directly binds to SARS-CoV-2 ORF3a and disrupts its canonical function in inhibiting autophagosome-lysosome fusion. This interaction restored lysosomal acidification and normalized perinuclear LAMP-1 localization, significantly impairing virion assembly as evidenced by disrupted nucleocapsid-RNA association and reduced intracellular viral titers. Additionally, SB431542 downregulated the CLEAR network genes responsible for lysosomal biogenesis, further restricting viral egress pathways. Our temporal analyses revealed that at later infection stages (36–48 hours post-infection [hpi]), SARS-CoV-2 exploits TGF-β-induced lysosomal membrane permeabilization (LMP) and apoptosis for viral release—processes effectively inhibited by SB431542 through suppression of GADD45b and BAX expression. These multiple mechanisms resulted in an exceptional EC_50_ of 751.8 nM against SARS-CoV-2. *In vivo* efficacy was demonstrated in embryonated chicken eggs, where SB431542 conferred dose-dependent protection against lethal infectious bronchitis virus (IBV) challenge, with a favorable therapeutic index of 34.54. Remarkably, sequential passaging of SARS-CoV-2 for 50 generations under SB431542 selection pressure failed to generate resistant variants, contrasting sharply with the rapid resistance emergence typical of direct-acting antivirals. These findings establish SB431542 as a promising broad-spectrum coronavirus inhibitor with a unique triple-mechanism approach that simultaneously targets viral entry via TGF-β/Smad modulation, disrupts ORF3a-mediated lysosomal dysfunction affecting assembly, and attenuates TGF-β-induced apoptosis during late-stage infection, collectively imposing multiple selective constraints that impede escape mutation development.

**IMPORTANCE:**

The COVID-19 pandemic highlighted the urgent need for antiviral drugs with high barriers to resistance. This study reveals that SB431542, a drug previously developed to inhibit TGF-β signaling, exhibits remarkable effectiveness against SARS-CoV-2 through an unprecedented triple-mechanism approach. Unlike conventional antivirals that target a single viral component, SB431542 simultaneously disrupts viral entry, assembly, and release by binding to the viral ORF3a protein and modulating host cellular processes. Most importantly, SARS-CoV-2 failed to develop resistance against SB431542 even after 50 generations of exposure—a significant advantage over current therapeutics that quickly lose effectiveness due to viral mutations. Our findings also uncover that coronaviruses exploit both lysosomal dysfunction and programmed cell death to spread efficiently, providing new targets for therapeutic intervention. This research establishes SB431542 as a promising broad-spectrum coronavirus inhibitor and demonstrates the value of targeting host-virus interactions to overcome antiviral resistance.

## INTRODUCTION

The coronavirus disease 2019 (COVID-19) pandemic, caused by severe acute respiratory syndrome coronavirus 2 (SARS-CoV-2), precipitated an unprecedented global health crisis, necessitating the development of antiviral therapeutics with high barriers to resistance. SARS-CoV-2 infection cascade begins when the spike (S) glycoprotein engages the angiotensin-converting enzyme 2 (ACE2) receptor, followed by proteolytic priming via host proteases, including TMPRSS2 and cathepsins, facilitating membrane fusion and viral genome release (1). Unlike many enveloped viruses that utilize the conventional secretory pathway, SARS-CoV-2 distinctively exploits lysosomal exocytosis for virion release—a mechanism differentiating it from other coronaviruses (2, 3).

The viral accessory protein ORF3a serves as a master regulator of this lysosomal subversion process (2). ORF3a inhibits autophagosome-lysosome fusion while simultaneously promoting lysosomal deacidification and exocytosis, thereby creating an environment conducive to virion preservation and efficient egress (2, 3).

This strategic manipulation of cellular degradation pathways not only facilitates viral dissemination but also compromises innate immune responses and cellular homeostasis (4, 5). The centrality of lysosomal dysregulation in SARS-CoV-2 pathogenesis suggests that targeting these mechanisms could yield promising therapeutic outcomes (6, 7).

In addition to lysosomal dysregulation, SARS-CoV-2 induces profound alterations in host signaling networks, particularly the transforming growth factor-beta (TGF-β) pathway, a pleiotropic cascade that regulates autophagy, apoptosis, and inflammatory responses (8–13). Hyperactivation of TGF-β/Smad signaling during SARS-CoV-2 infection enhances furin-mediated spike protein processing and cell-cell fusion events critical for viral propagation (8, 14). Additionally, dysregulated TGF-β signaling contributes to the cytokine storms and pulmonary fibrosis characteristic of severe COVID-19 (15–17). While elevated apoptotic indices have been documented in lung specimens from fatal COVID-19 cases, the mechanistic relationship between TGF-β-induced apoptosis and SARS-CoV-2 replication dynamics remains poorly studied (16, 18–20).

SB431542 is a potent and selective inhibitor of activin receptor-like kinase 5 (ALK5), a key mediator of TGF-β signaling (21). In this study, we demonstrate that SB431542 exhibits antiviral potential against multiple coronaviruses. By using a combination of *in vitro*, *in silico*, and *in vivo* models, our findings reveal that beyond its canonical inhibition of TGF-β/Smad signaling, SB431542 exhibits previously unrecognized activity against SARS-CoV-2 through direct interaction with the viral ORF3a protein, thereby restoring lysosomal function. Additionally, we uncovered a temporal dimension to SARS-CoV-2 egress mechanisms, wherein, besides lysosomal exocytosis, the virus exploits apoptotic pathways during late-stage infection, a process effectively disrupted by SB431542. These mechanisms significantly reduce SARS-CoV-2-mediated cytopathic effects (CPE) in Vero cells while preventing infectious bronchitis virus (IBV)-induced stunted growth in embryonated chicken eggs.

A perennial challenge in antiviral therapeutics is the emergence of drug-resistant escape mutants under prolonged selection pressure (22–25). Resistance mutations often compromise drug efficacy and limit long-term therapeutic utility (26, 27). Besides antiviral mechanistic insights, we also addressed this issue by subjecting SARS-CoV-2 to 50 serial passages under SB431542 pressure. Notably, no resistant mutants were observed, underscoring SB431542’s high genetic barrier to resistance.

Collectively, our findings highlight SB431542 as a potential therapeutic candidate with multiple antiviral mechanisms, targeting both host (TGF-β signaling) and viral (ORF3a-mediated lysosomal dysfunction) components, with a remarkably high genetic barrier to resistance.

## RESULTS

### SB431542 suppresses SARS-CoV-2 entry and release from Vero cells during the early hours of infection

**Fig 1:**
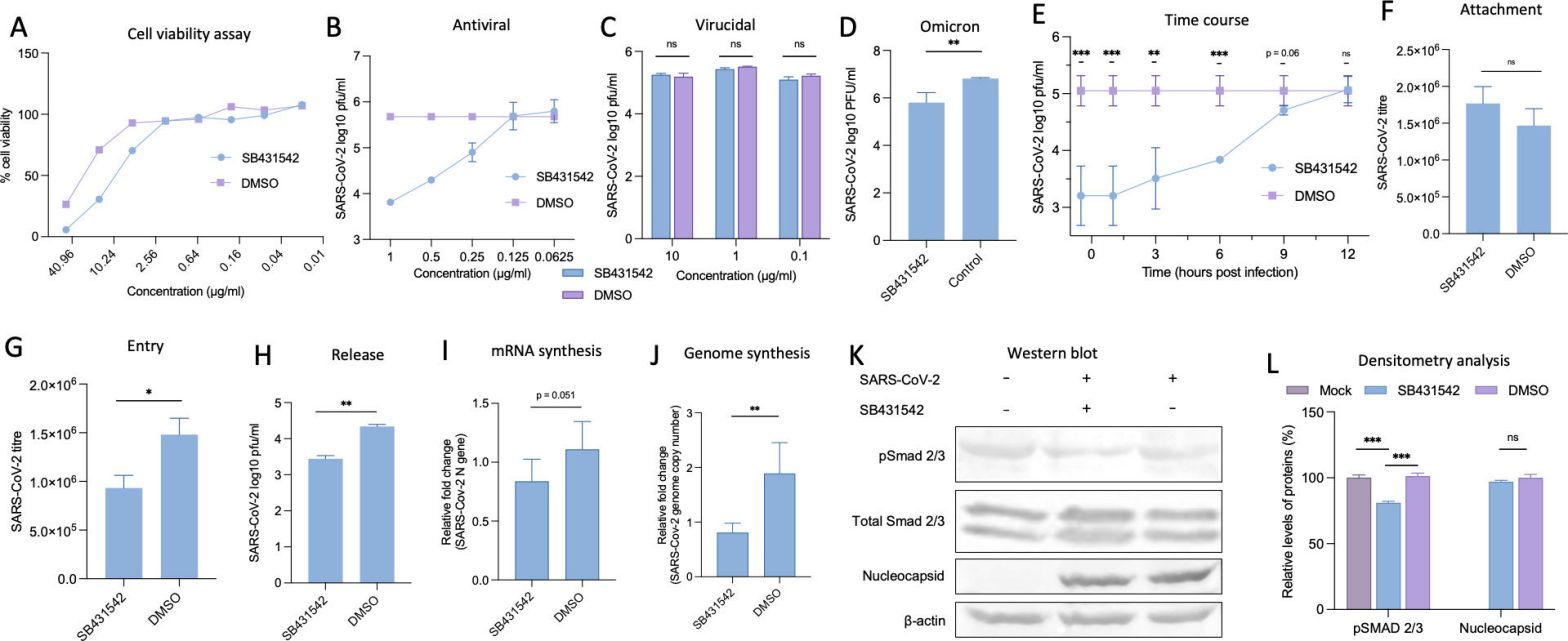
SB431542 treatment suppresses SARS-CoV-2 replication. (**A**) Cytotoxicity of SB431542 (MTT assay): Vero cells were treated with the indicated concentrations of SB431542 or equivalent volumes of dimethyl sulfoxide (DMSO) in triplicate for 96 hours. Cell viability was measured using the MTT assay, and the CC50 was calculated using the Reed-Muench method. (**B**) *In vitro* anti-SARS-CoV-2 efficacy: Vero cells were infected with SARS-CoV-2 wild-type variant at a multiplicity of infection (MOI) of 0.1 and treated with the indicated concentration of SB431542 or DMSO in triplicate. Infectious virus particles in the culture supernatants were quantified at 24 hours post-infection (hpi) by plaque. (**C**) Virucidal activity: SARS-CoV-2 wild type was incubated with the indicated concentrations of SB431542 or equivalent volumes of DMSO for 90 minutes at 37°C. The virus-inhibitor mixture was diluted (1:1,000), and residual viral infectivity was determined by plaque assay. (**D**) *In vitro* anti-SARS-CoV-2 efficacy: Vero cells were infected with SARS-CoV-2 omicron variant at an MOI of 0.1 and treated with 1 µg/mL SB431542 or DMSO in triplicate. Infectious virus particles in the culture supernatants were quantified at 24 hpi by plaque assay. (**E**) Time-of-addition assay: confluent monolayers of Vero cells were infected with SB431542 at an MOI of 5, washed with phosphate-buffered saline (PBS), and treated with SB431542 or DMSO at indicated time points. Supernatants were collected at 12 hpi, and infectious virions were quantified by plaque assay. (**F**) Attachment assay: Vero cells, in triplicate, were preincubated with SB431542 or DMSO for 1 hour before infection with SARS-CoV-2 wild type at an MOI of 5 for 1 hour at 4°C to allow attachment. Cells were then washed five times with PBS, lysed by rapid freeze-thaw, and the virus released was quantified by plaque assay. (**G**) Entry assay: Vero cells, in triplicate, were infected with SARS-CoV-2 wild type at an MOI of 5 for 1 hour at 4°C in SB431542-free medium to allow attachment. After washing five times with ice-cold PBS, fresh medium containing SB431542 or DMSO was added, and cells were incubated at 37°C for 1 hour to permit viral entry. Cells were subsequently washed and incubated with SB431542-free Dulbecco’s modified Eagle medium (DMEM). Virus particles released at 12 hpi were titrated by plaque assay. (**H**) Virus release assay: Vero cells, in triplicate, were infected with SARS-CoV-2 wild type at an MOI of 5 for 1 hour, washed five times with PBS, and supplemented with fresh DMEM. At 8 hpi, when virus budding presumably begins, cells were washed again and fresh medium containing SB431542 or DMSO was added. Virus particles released into the supernatant at 4 hours post-drug treatment were quantified by plaque assay. (**I, J**) Viral mRNA/genome synthesis: Vero cells were infected with SARS-CoV-2 wild type at an MOI of 5, washed with PBS, and treated with SB431542 or DMSO at 2 hpi. RNA and DNA were isolated from cells harvested at 6 hpi. For mRNA quantification, cDNA was synthesized from RNA using oligo(dT) primers. SARS-CoV-2 *N* gene expression was quantified by quantitative real-time PCR (qRT-PCR), normalized to β-actin (housekeeping control), and analyzed using the ∆∆Ct method. (**K**) Western blot analysis: Vero cells were infected with SARS-CoV-2 wild type and treated with SB431542 or vehicle control at 2 hpi. At 12 hpi, cell lysates were prepared in RIPA buffer and analyzed by western blot using anti-pSMAD2/3, anti-total SMAD2/3, anti-SARS-CoV-2 Nucleocapsid, or GAPDH antibody. (**L**) Relative band intensities were quantified using ImageJ software. pSMAD2/3 levels were normalized to total SMAD2/3, and Nucleocapsid levels were normalized to GAPDH as loading controls. All values represent means ± SD from at least three independent experiments. Statistical significance was determined using Student’s *t*-test (ns, non-significant; *P < 0.05; **P < 0.01; ***P < 0.001).

In a comprehensive screening of approximately 150 host kinase inhibitors, we identified TGF-β receptor I (ALK5) antagonist SB431542 as a potent inhibitor of SARS-CoV-2 replication. SB431542 exhibited dose-dependent antiviral efficacy in Vero cells at non-cytotoxic concentrations (Fig. 1A), with an EC_50_ of 751.8 nM (Fig. 1B). This potency substantially exceeds that of the FDA-approved nucleoside analog remdesivir (EC_50_: 1.65 µM) in comparable cellular systems (28), positioning SB431542 as a potent inhibitor against SARS-CoV-2.

To delineate whether SB431542 directly neutralizes extracellular virus, we conducted virucidal assays wherein SARS-CoV-2 wild type was pre-incubated with SB431542 at concentrations up to 10-fold above the non-cytotoxic threshold. The absence of reduced infectivity (Fig. 1C) indicated that SB431542 does not compromise virion integrity directly but likely targets intracellular replication processes. Importantly, the antiviral efficacy of SB431542 was also confirmed against the Omicron variant, supporting its potential as a pan-coronavirus inhibitor (Fig. 1D).

To further elucidate the specific stages of the SARS-CoV-2 life cycle affected by SB431542, we performed a time-of-addition assay by infecting Vero cells at a multiplicity of infection (MOI) of 5 and adding the inhibitor at various time points post-infection. Addition of SB431542 either 30 minutes pre-infection or within the first hour post-infection (hpi) conferred maximal inhibition, with a gradual attenuation of efficacy when added at later time points (Fig. 1E). This temporal sensitivity pattern suggested interference with both early viral entry mechanisms and late-stage post-replicative processes.

To precisely elucidate the specific phases of the viral lifecycle targeted by SB431542, we performed stage-specific assays. Temperature-restricted (4°C) attachment experiments demonstrated that SB431542 does not significantly impair the initial virion-receptor engagement (Fig. 1F). However, when pre-attached virions were permitted to internalize by temperature elevation to 37°C, SB431542 treatment significantly suppressed productive infection (Fig. 1G), indicating disruption of post-attachment entry mechanisms. Additionally, when administered at 12 hpi following the removal of pre-released virions by extensive washing, SB431542 significantly reduced subsequent virion egress (Fig. 1H), establishing its capacity to inhibit late-stage virion release.

Importantly, when SB431542 was applied during the intermediate phase (3 hpi), neither intracellular viral RNA accumulation (Fig. 1J) nor viral protein synthesis (Fig. 1K) was appreciably affected, while phosphorylation of Smad2/3, the canonical mediator of TGF-β signaling, was markedly attenuated (29, 30).

Taken together, these results indicate that SB431542 inhibits SARS-CoV-2 entry and release without affecting other steps such as RNA or protein synthesis. While its effect on viral entry has been previously reported (8), our findings highlight its additional role in disrupting egress mechanisms.

### SB431542 restores lysosomal function by subverting ORF3a

SARS-CoV-2 uniquely exploits lysosomal exocytosis for viral egress, a process orchestrated by its accessory protein ORF3a (2, 3). Unlike its counterpart in SARS-CoV, the SARS-CoV-2 ORF3a protein inhibits autophagosome-lysosome fusion while simultaneously promoting lysosomal deacidification, thereby creating an environment conducive to virion preservation and efficient release (2, 31). To investigate whether SB431542 targets this pathway, we examined autophagosome dynamics using GFP-LC3 as a marker for autophagosome accumulation (32).

**Fig 2:**
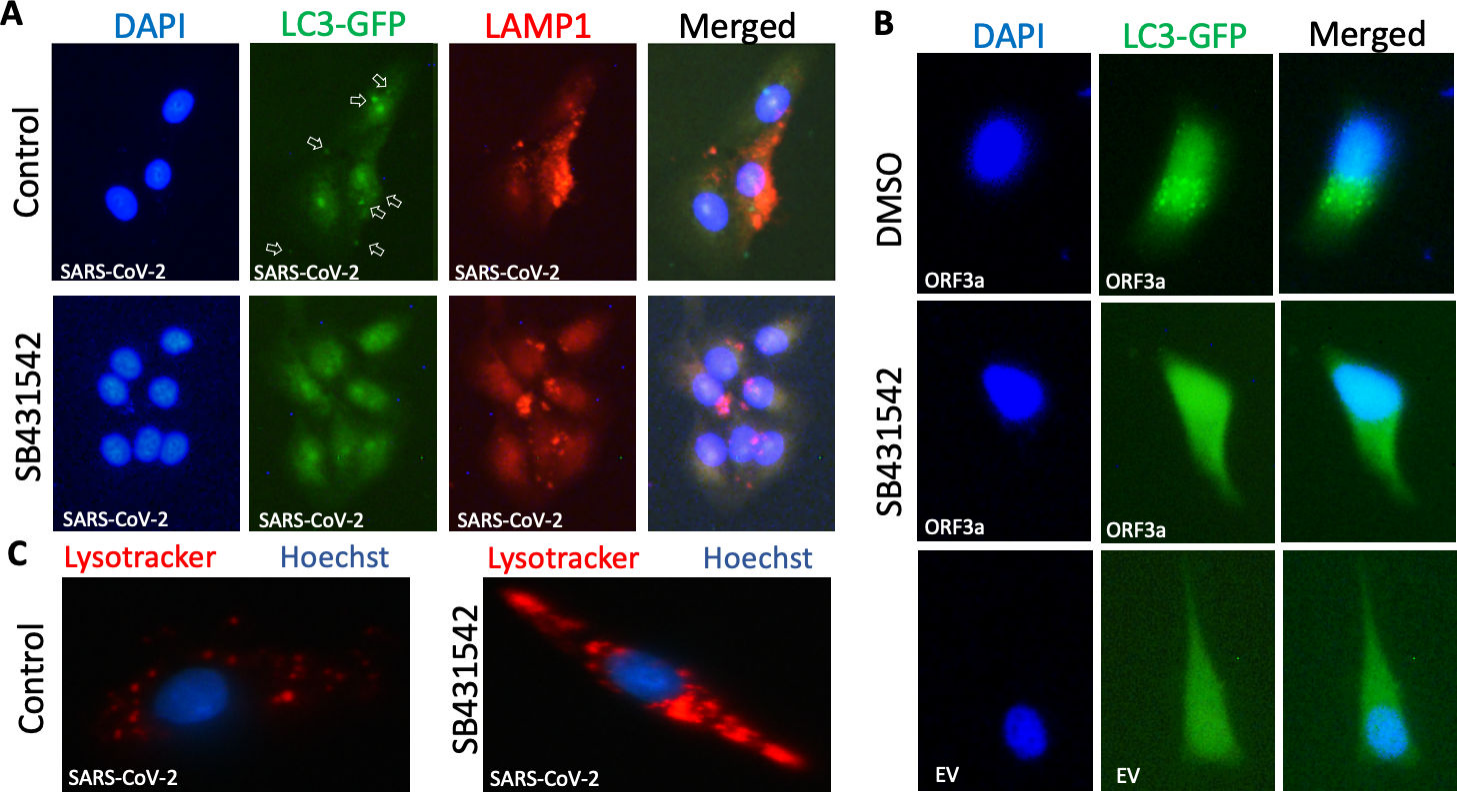
SB431542 disrupts ORF3a-mediated lysosomal dysregulation. Immunofluorescence imaging of (**A**) Vero cells transfected with LC3-GFP followed by SARS-CoV-2 wild-type infection: Vero cells were transfected with LC3-GFP. After 48 hours, SARS-CoV-2 wild type was inoculated at an MOI of 5 for 1 hour, followed by washing with PBS and incubation for 12 hours with SB431542 (lower panel) or DMSO (upper panel). At 12 hpi, cells were fixed and permeabilized, followed by probing with anti-LAMP1 antibody and DAPI. (**B**) HeLa cells transfected with LC3-GFP: HeLa cells were transfected with LC3-GFP and SARS-CoV-2 ORF3a or empty vector for 48 hours and treated with SB431542 or vehicle control, followed by fixing and permeabilization. DAPI was used to stain the nucleus. (**C**) Lysotracker assay: Vero cells were infected with SARS-CoV-2 wild type at 1 MOI for 1 hour, followed by treatment with DMSO (left panel) or SB431542 (right panel). At 24 hpi, cells were stained with Lysotracker and Hoechst dyes and analyzed with a fluorescent microscope.

Consistent with previous studies, overexpression of GFP-LC3 followed by SARS-CoV-2 infection led to cytoplasmic foci formation indicative of autophagosome accumulation (5, 31) (Fig. 2A). However, treatment with SB431542 inhibited this aggregation and dispersed GFP-LC3 throughout the cytoplasm. This effect was further validated in HeLa cells overexpressing ORF3a and GFP-LC3, wherein treatment with SB431542 significantly reduced LC3-GFP puncta formation (Fig. 2B). These observations suggest that SB431542 interferes with ORF3a-mediated disruption of autophagosome-lysosome fusion. It is noteworthy that TGF-β1 signaling is well-established in autophagic induction (15, 33); the apparent autophagy-inducing effect of its putative inhibitor, SB431542, in the context of SARS-CoV-2 infection was intriguing.

### SB431542 targets SARS-CoV-2 ORF3a at the Saposin-A interaction interface

To elucidate the potential mechanism underlying this phenomenon, we conducted a literature survey and found that ORF3a is a potential target of macro-heterocyclic compounds, such as chlorin (34). This prompted us to perform in-depth computational analyses, including protein-ligand structural modeling and molecular dynamics simulations.

**Fig 3:**
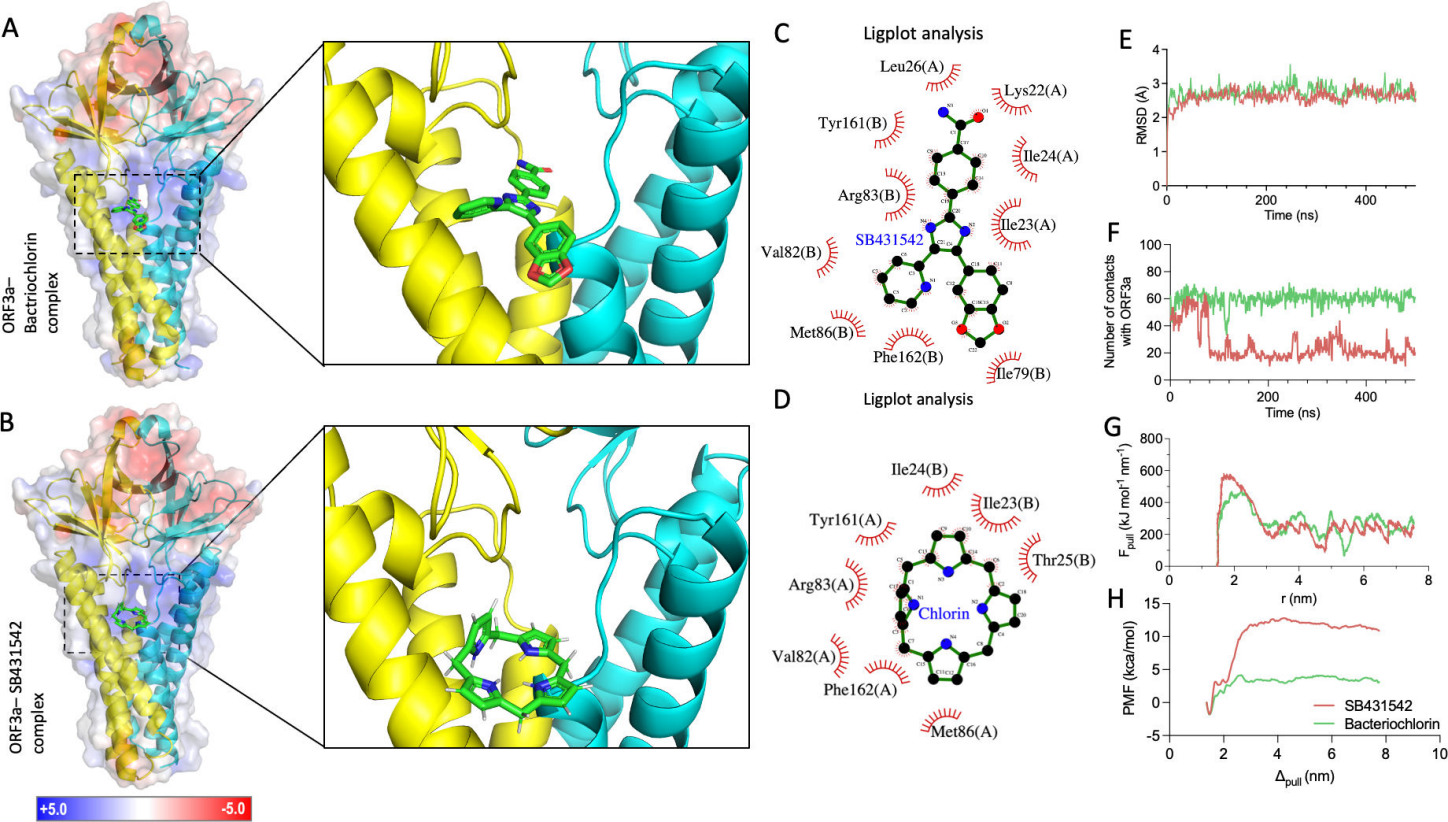
SB431542 binds to ORF3a via off-target effect. (**A**) ORF3a–SB431542 complex (Boltz prediction): an illustration of the Boltz-generated ORF3a–SB431542 structure is shown. The ORF3a chains are colored in yellow and cyan, and a color-coded surface plot of the electrostatics is shown as well. The side shows a zoom into the SB431542 binding region. (**B**) ORF3a–Bacteriochlorin complex (Boltz prediction): an illustration of the Boltz-generated ORF3a–Bacteriochlorin structure is shown. The ORF3a chains are colored in yellow and cyan, and a color-coded surface plot of the electrostatics is shown as well. The side shows a zoom into the Bacteriochlorin binding region. (**C, D**) ORF3a–ligand interactions: Liglot analysis of SB431542 (**C**) and bacteriochlorin (**D**) bound to ORF3a. (**E**) Root mean square deviation (RMSD) of protein complexes: the root mean square fluctuation of the ORF3a complexes from both performed 500 ns molecular dynamics (MD) simulations is plotted over the simulated time range. Though fluctuations are visible, a general stabilization between 2 and 3 Å is visible. (**F**) Number of protein-ligand contacts over time: here, the absolute number of contacts based on a 4 Å cutoff between the ligand and ORF3a dimer is illustrated over simulated time. While the number of contacts oscillates at around 60 contacts for Bacteriochlorin, a drop to around 20 contacts is visible at 100 ns. (**G**) Non-equilibrium pulling force: the pulling force observed in the non-equilibrium pulling simulation is illustrated as a function of simulation time for both ORF3a-SB431542 and ORFa-Bacteriochlorin complexes. An overall higher peak of to-be-overcome force can be observed for SB431542 (583 kJ/mol/nm) compared to Bacteriochlorin (470 kJ/mol/nm). (**H**) Potential of mean forces profile: the two generated potential of mean forces profiles are illustrated over the pulling distance for both investigated ligands. After a first decrease in potential of mean force (PMF), both curves continuously increase until reaching a plateau at around 4 nm. While the absolute PMF value shows a slight downward trend from 4 nm on the absolute difference toward the minimal PMF value is significantly higher, implying a stronger ΔG value.

Using the diffusion-based structure prediction tool Boltz (35), we generated protein-ligand complexes based on the resolved ORF3a dimer structure (PDB ID: 6XDC). The modeling revealed that SB431542 binds to a specific cavity within the ORF3a dimer (Fig. 3A). As a comparative control, we modeled the binding of bacteriochlorin, a structural analog of chlorin previously reported to interact with ORF3a (Fig. 3B) (34). A detailed examination via Ligplot analysis (36) of the protein-ligand interfaces demonstrated that both compounds established interactions with similar residues in the ORF3a dimer (Fig. 3C and D).

Molecular dynamics simulations (500 ns) demonstrated remarkable stability of both protein-ligand complexes, as evidenced by minimal root mean square deviation (RMSD) fluctuations throughout the simulation period (Fig. 3E). Additional analyses, including root mean square fluctuation (RMSF) and radius of gyration (Rg), further confirmed the structural integrity of the complexes during simulation (Fig. S1A and B). Quantitative assessment of protein-ligand interactions revealed distinct binding characteristics. While bacteriochlorin maintained approximately 60 contacts with ORF3a throughout the simulation, SB431542 exhibited fewer contacts (approximately 20–30) during equilibrium phases (Fig. 3F). Despite this difference in contact profiles, both compounds adopted similar binding modes within the ORF3a structure (Fig. S1C).

To understand the interaction dynamics and binding affinity, we first performed MM-GBSA analysis (37, 38), which revealed slightly lower average interaction energy for ORF3a-SB431542 compared to ORF3a-bacteriochlorin (Fig. S1D). This prompted us to perform non-equilibrium pulling steered molecular dynamics, which demonstrated that SB431542 required significantly greater force for extraction from its binding pocket compared to bacteriochlorin (Fig. 3G), suggesting stronger binding interactions despite maintaining fewer contacts.

The more rigorous umbrella sampling approach further provided definitive evidence of the superior binding affinity of SB431542 (39). Analysis of the potential of mean force (PMF) between protein and ligand, illustrated in Fig. 3H, revealed substantially stronger energetic differences for SB431542 compared to bacteriochlorin. Calculating all possible differences and using the mean values (Fig. S1E), we determined binding free energies of ΔG = −13.32 kcal/mol for SB431542 and ΔG = −5.28 kcal/mol for bacteriochlorin.

To substantiate the binding potential of SB431542-ORF3a, we conducted an isothermal titration calorimetry (ITC) assay. Direct binding measurements demonstrated that SB431542 interacts with ORF3a with a dissociation constant (K_D_) of 220 ± 68.1 nM at *n* = 2.36 ± 0.108 sites, indicating a favorable binding affinity suitable for therapeutic intervention. The binding was characterized by unfavorable enthalpy (ΔH = 34.3 ± 0.522 kcal/mol), leading to endothermic peaks and highly favorable entropy (TΔS = 43.4 kcal/mol), yielding an overall binding free energy (ΔG) of −9.08 kcal/mol. This thermodynamic signature is characteristic of entropy-driven binding, typically associated with hydrophobic interactions, desolvation effects, and conformational flexibility upon ligand binding. The endothermic nature of the binding, coupled with the favorable entropy change, suggests that SB431542 binding involves favorable hydrophobic contacts with ORF3a, consistent with our molecular dynamics simulations showing its preference for hydrophobic residues within the binding pocket. Importantly, the experimental binding affinity (K_D_ = 220 ± 68.1 nM) fairly correlates with the antiviral efficacy (EC_50_ = 751.8 nM) observed in cellular assays, supporting a direct mechanistic relationship between ORF3a binding and viral inhibition. While the computational binding free energy (ΔG = −13.32 kcal/mol) appears slightly more favorable than experimental values, this discrepancy is typical when comparing umbrella sampling calculations to ITC measurements due to differences in accounting for solvent entropy and protein dynamics.

Intriguingly, structural alignment with the recently published ORF3a-Saposin-A complex (PDB ID: 8EQU) revealed that the SB431542 binding site directly overlaps with the protein-protein interaction interface between ORF3a and Saposin-A (40) (Fig. S1H). Higher magnification views of both the initial and final states of the molecular dynamics (MD) simulation demonstrated that SB431542 occupies the precise interface where critical interactions between ORF3a and Saposin-A occur (Fig. S1I and J). This structural insight provides a mechanistic explanation for the ability of SB431542 to inhibit lysosomal exocytosis pathways. Together, these computational findings, corroborated by experimental ITC validation, demonstrate that SB431542 disrupts ORF3a-mediated lysosomal dysfunction through direct protein binding, thereby inhibiting SARS-CoV-2 egress.

### SB431542 treatment restores lysosomal pH

Besides LC3, we also examined lysosomal function by analyzing LAMP-1 localization, a key lysosomal membrane protein involved in lysosomal biogenesis, autophagy, and SARS-CoV-2 exocytosis (2, 41, 42). In SARS-CoV-2 wild-type-infected cells, LAMP-1 predominantly localized to peripheral regions, indicative of active lysosomal exocytosis (2) (Fig. 2A). In contrast, SB431542 treatment sequestered LAMP-1 within perinuclear regions. Additionally, SB431542-treated cells exhibited lower LAMP-1 expression compared to vehicle controls, suggesting inhibition of the canonical ORF3a function that typically increases LAMP-1 expression following SARS-CoV-2 infection (2, 31).

Given that peripheral lysosomes are less acidic than juxtanuclear ones (43), we tracked the lysosomes using lysotracker dye, which gives an approximation of both location and pH of lysosomes. As expected, SARS-CoV-2 wild-type-infected cells displayed peripheral lysosome localization with diminished fluorescence intensity, indicative of deacidification and active exocytosis (Fig. 2C). Treatment with SB431542 restored fluorescence intensity and redistributed lysosomes to perinuclear regions, indicating restoration of lysosomal acidification. These evidences collectively suggest that SB431542 treatment restored lysosomal function by targeting ORF3a.

### SB431542 disrupts SARS-CoV-2 assembly

**Fig 4:**
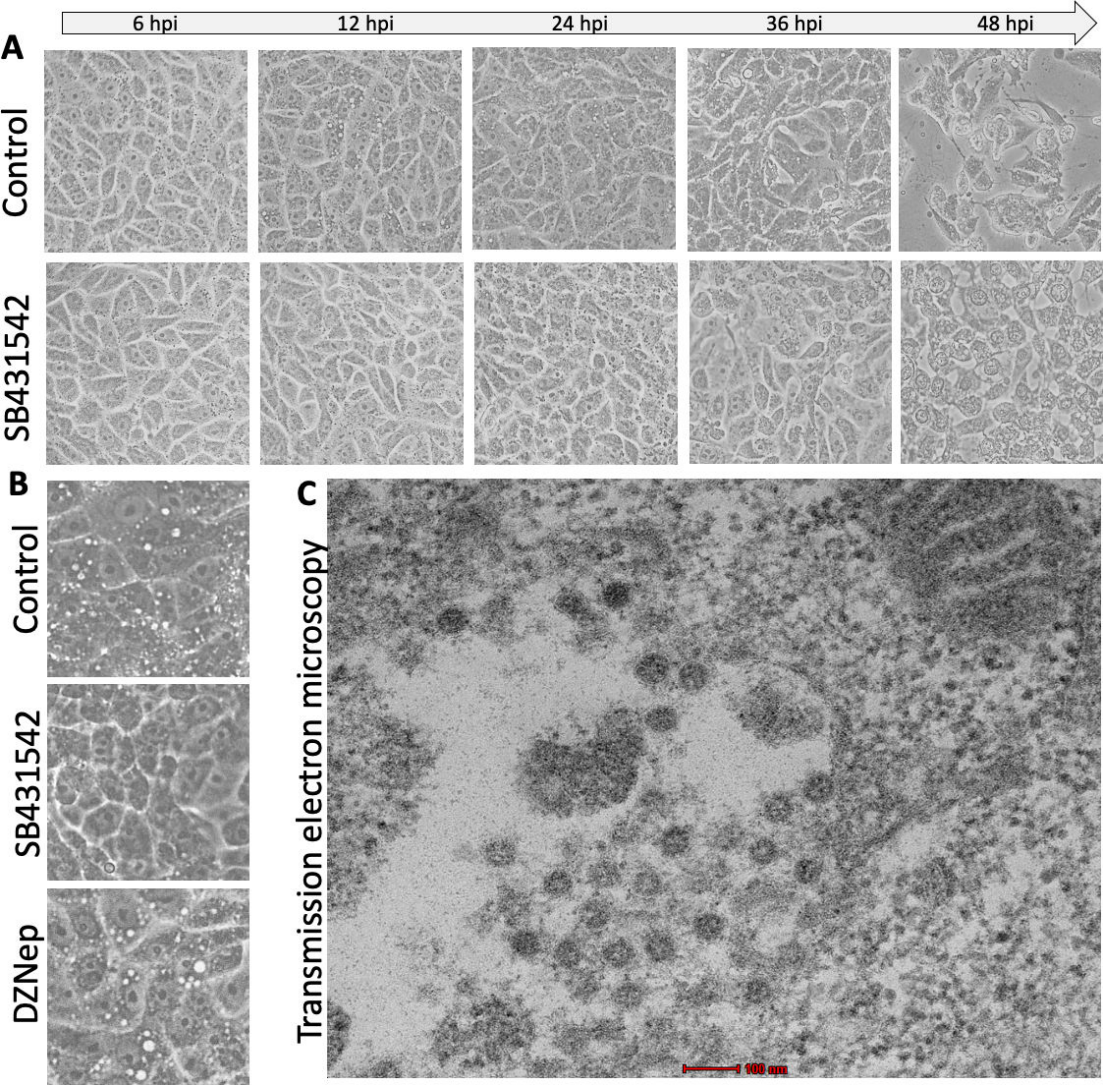
SB431542 disrupts SARS-CoV-2-mediated lysosomal vacuolization. (**A**) Bright field microscopy: Vero cells were infected with SARS-CoV-2 wild type at 5 MOI for 1 hour, followed by treatment with DMSO (upper panel) or SB431542 (lower panel). Images were taken at the indicated time points. (**B**) Phase contrast microscopy: Vero cells were infected with SARS-CoV-2 wild type at 5 MOI for 1 hour, followed by treatment with DMSO (upper panel), SB431542 (middle panel), or DZNep (lower panel). Images were taken at 12 hpi. (**C**) Transmission electron microscopy (TEM): Vero cells were infected with SARS-CoV-2 wild type at 5 MOI for 1 hour. At 12 hpi, cells were fixed using glutaraldehyde solution, and subsequently, blocks were prepared and thin sectioning was performed for TEM imaging.

To investigate the functional consequences of SB431542-mediated restoration of lysosomal function on SARS-CoV-2 egress, we performed several morphological and biochemical analyses. Bright-field microscopy revealed pronounced differences in cellular architecture between control and SB431542-treated infected cells at 12 hpi. Control-infected cells exhibited substantial lysosomal vacuolization (Fig. 4A, upper panel), with particularly prominent vacuolar structures visible under phase contrast (Fig. 4B, upper panel).

SB431542 treatment remarkably suppressed this vacuolization phenotype (Fig. 4A, lower panel; Fig. 4B, middle panel). We also examined the effect of DZNep, another SARS-CoV-2 inhibitor with a distinct mechanism of action (44), which failed to prevent vacuolization (Fig. 4B, lower panel), indicating the specificity of the action of SB431542. Transmission electron microscopy (TEM) analysis further confirmed that these vacuoles contained assembled SARS-CoV-2 virions (Fig. 4C), implicating lysosomal dysfunction and subsequent vacuolization in viral assembly and release (41).

**Fig 5:**
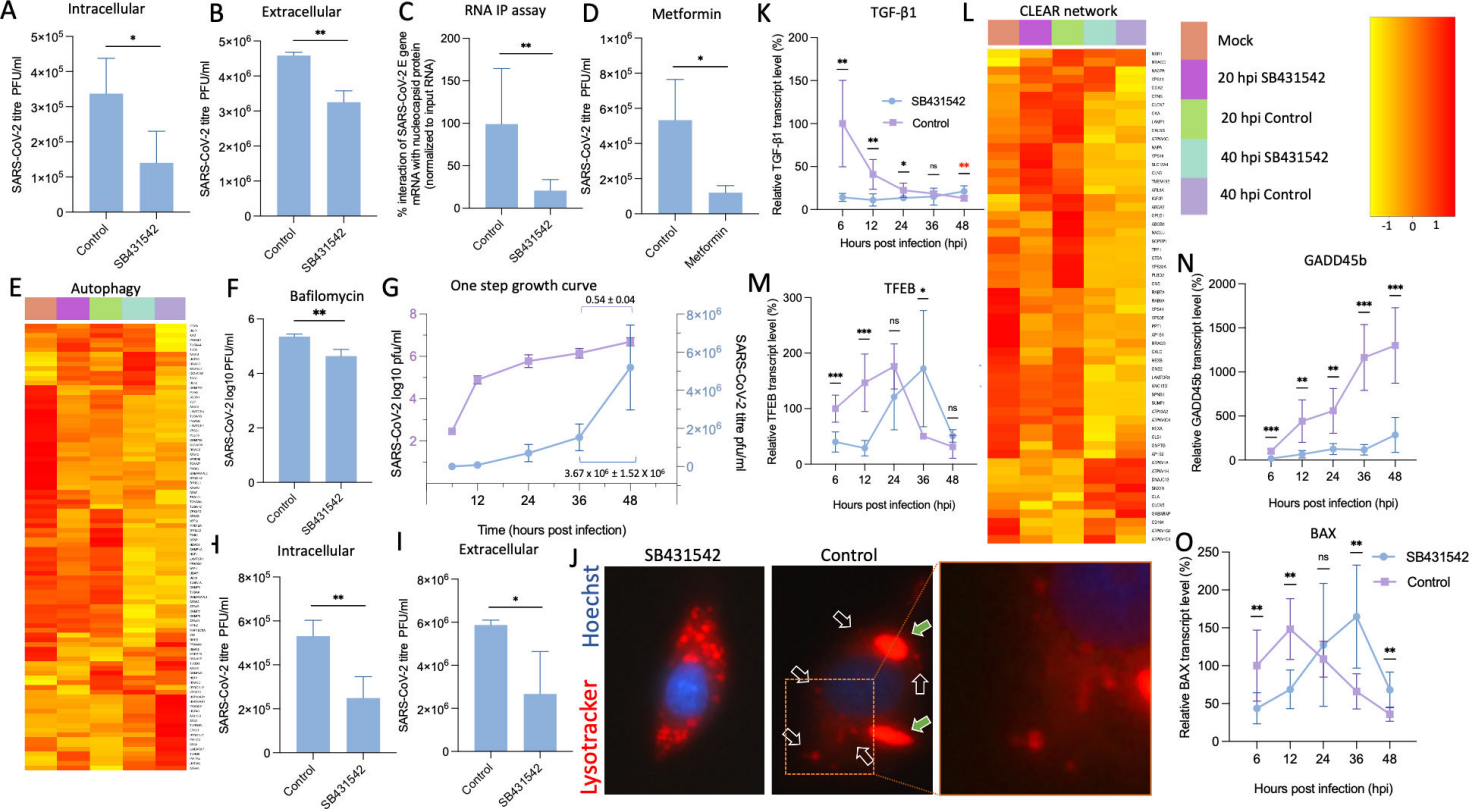
SB431542 blocks SARS-CoV-2 release. (**A, B**) Quantification of intra- and extracellular virions at 24 hpi: Vero cells were infected with SARS-CoV-2 wild type at 1 MOI for 1 hour, followed by treatment with DMSO or SB431542. At 24 hpi, supernatants were harvested and cells were washed with PBS multiple times, followed by rapid freeze-thaw cycles to retrieve intracellular virions. Infectious virions from supernatant and cell lysates were quantified with a plaque assay. (**C**) RNA immunoprecipitation (RNA-IP) assay: Vero cells were infected with SARS-CoV-2 wild type at an MOI of 5 and treated with SB431542 or DMSO at 2 hpi. At 16 hpi, cell lysates were prepared for RNA-IP as described in Materials and Methods. Lysates were incubated with α-nucleocapsid (reactive antibody), α-ERK (non-reactive antibody), or IP buffer alone (beads control), followed by Protein A Sepharose slurry incubation. After washing and cross-link reversal, cDNA was obtained from immunoprecipitated RNA, and SARS-CoV-2 RNA (N gene) was quantified by qRT-PCR and normalized to input controls. (**D**) Metformin antiviral assay: Vero cells were infected with SARS-CoV-2 wild type at 0.1 MOI for 1 hour, followed by treatment with metformin or vehicle control. At 24 hpi, supernatants were harvested and viruses were quantified using the plaque assay. (**E**) RNAseq analysis of autophagy-related genes: Vero cells were infected with SARS-CoV-2 wild type at 5 MOI for 1 hour, followed by treatment with DMSO or SB431542. At indicated time points, cells were scraped, and total RNA was isolated, followed by RNAseq analysis as mentioned in Materials and Methods. For analysis of autophagy-related genes from samples, KEGG pathway filtering was utilized. (**F**) Bafilomycin antiviral assay: Vero cells were infected with SARS-CoV-2 wild type at 0.1 MOI for 1 hour, followed by treatment with bafilomycin or vehicle control. At 24 hpi, supernatants were harvested and viruses were quantified using the plaque assay. (**G**) One-step growth curve analysis. Vero cells were infected with SARS-CoV-2 wild type at 1 MOI for 1 hour, followed by washing with PBS and supplemented with fresh DMEM. Supernatants were harvested at the indicated time points, and the virus was quantified using a plaque assay. (**H, I**) Quantification of intra- and extracellular virions at 48 hpi: Vero cells were infected with SARS-CoV-2 wild type at 1 MOI for 1 hour, followed by treatment with DMSO or SB431542. At 48 hpi, supernatants were harvested, and cells were washed with PBS multiple times, followed by rapid freeze-thaw cycles to retrieve intracellular virions. Infectious virions from supernatant and cell lysates were quantified with a plaque assay. (**J**) Lysotracker assay: Vero cells were infected with SARS-CoV-2 wild type at 1 MOI for 1 hour, followed by treatment with DMSO (left panel) or SB431542 (right panel). At 42 hpi, cells were stained with Lysotracker and Hoechst dyes and analyzed with a fluorescent microscope. (**K**) Quantification of TGF-β1 gene: Vero cells were infected with SARS-CoV-2 wild type at 1 MOI for 1 hour, followed by treatment with DMSO or SB431542. At indicated time points, cells were scraped and RNA was isolated, followed by cDNA library preparation using oligo(dT). TGF-β1 genes were amplified from the harvested cells and normalized to the β-actin gene (housekeeping control). Relative fold change was calculated using the ∆∆Ct method. (**L**) RNAseq analysis of CLEAR network-related genes: Vero cells were infected with SARS-CoV-2 wild type at 5 MOI for 1 hour, followed by treatment with DMSO or SB431542. At indicated time points, cells were scraped, and total RNA was isolated, followed by RNAseq analysis as mentioned in Materials and Methods. For analysis of CLEAR network-related genes from samples, KEGG pathway filtering was utilized. (**M, N, and O**) Quantification of GADD45b, TFEB, and BAX genes: Vero cells were infected with SARS-CoV-2 wild type at 1 MOI for 1 hour, followed by treatment with DMSO or SB431542. At indicated time points, cells were scraped and RNA was isolated, followed by cDNA library preparation using oligo(dT). GADD45b (**M**), TFEB (**N**), and (**O**) BAX genes were amplified from the harvested cells and normalized to the β-actin gene (housekeeping control). Relative fold change was calculated using the ∆∆Ct method. Values represent means ± SD from at least three independent experiments. Statistical significance was determined using Student’s *t*-test (ns, non-significant; *P < 0.05; **P < 0.01; ***P < 0.001).

To quantitatively assess the impact of SB431542 on virion assembly and egress, we conducted parallel analyses of intracellular and extracellular viral loads. Following infection at MOI 1, SB431542 treatment significantly reduced both intracellular virion abundance (Fig. 5A) and extracellular viral titers (Fig. 5B) at 24 hpi. This concurrent diminution of both intracellular and extracellular viral populations suggested that SB431542 disrupts virion biogenesis upstream of the egress process. Combined with data from Fig. 2, these results suggest that SB431542-mediated lysosomal acidification disrupts the production of infectious virions.

The nucleocapsid (N) protein plays a central role in coronavirus assembly by encapsidating viral genomic RNA (45, 46). RNA immunoprecipitation assays using anti-N antibodies revealed that SB431542 treatment significantly abrogated the association between N protein and viral RNA (Fig. 5C), indicating disruption of a critical initial step in virion assembly.

To further substantiate these findings, we probed the localization of viral genomic RNA (vgRNA) and N protein using J2 and anti-N antibodies, respectively. In untreated infected cells (Fig. S1, upper panel), the N protein exhibited diffuse cytoplasmic distribution, while vgRNA was predominantly clustered in the perinuclear region—a pattern consistent with active virion assembly sites (47, 48). By contrast, SB431542 treatment led to a pronounced dispersion of vgRNA throughout the cytoplasm, with a loss of perinuclear clustering (Fig. S2, upper panel). This spatial redistribution (zoomed view), together with the RNA immunoprecipitation (RNA-IP) results, suggests that SB431542 disrupts the formation of perinuclear assembly platforms, likely by impeding the ability of N protein to efficiently package viral RNA.

Given the established role of lysosomal acidification in regulating autophagic processes (49), we hypothesized that the effect of SB431542 on viral assembly might be mechanistically linked to the restoration of autophagic flux. To test this, we employed metformin, an over-the-counter available drug known for autophagy inducer (50), which significantly reduced SARS-CoV-2 wild-type titers by 72.5% with EC_50_ ~35 µM at a non-cytotoxic dose of 50 µM (Fig. 5D), phenocopying the effect of SB431542. Transcriptomic analysis further revealed that SB431542 modulated the expression of autophagy-related genes at both early (20 hpi) and late (40 hpi) infection time points (Fig. 5E). Conversely, complete inhibition of autophagy using bafilomycin A1 also suppressed viral titers (Fig. 5F), consistent with previous studies demonstrating that SARS-CoV-2 requires induction of early autophagy while inhibiting later stages of autophagosome-lysosome fusion (51). Taken together, these findings demonstrate that SB431542 restores lysosomal acidification and autophagic flux by targeting ORF3a, thereby disrupting SARS-CoV-2 assembly.

### SB431542 suppresses SARS-CoV-2 infection through suppression of the canonical apoptosis pathway

While SARS-CoV-2 has a replication cycle of approximately 8 h (44, 52, 53), significant CPEs were only observed at 42–48 hpi even at high MOI of 5 (Fig. 4A). Prior to this time point, infected cell morphology remained comparable to uninfected controls, consistent with previous observations that viral egress does not compromise cell viability until later stages of infection (3). This observation is particularly noteworthy as most previous studies investigating SARS-CoV-2 egress mechanisms were temporally restricted to early infection stages (16–24 hpi), during which lysosomal exocytosis serves as the primary release mechanism (2, 3, 54).

To characterize viral replication dynamics during this extended infection period, we performed one-step growth curve analysis up to the onset of observable CPE. Between 36 and 48 hpi, we documented a substantial 340% increase in viral titers, equivalent to a linear-scale increment of 3.67 × 10^6^ ± 1.52 × 10^6^ (Fig. 5G). SB431542 treatment significantly suppressed both intracellular and extracellular viral titers even at 48 hpi (Fig. 5H and I).

Lysotracker staining at 42 hpi, a time point where CPE was starting to occur, revealed stress-induced lysosome membrane permeabilization (LMP) (55) in control-treated cells (green arrows in Fig. 5J), alongside numerous deacidified lysosomes (black arrows). These morphological changes are characteristic of cells undergoing apoptosis, suggesting that high cytosolic viral loads trigger LMP-mediated cell death pathways to facilitate late-stage viral release (55, 56). In contrast, SB431542-treated cells maintained acidic lysosomal compartments, correlating with reduced viral titers (Fig. 5J, right panel and zoomed view).

To determine whether this inhibitory effect is mediated through canonical TGF-β signaling inhibition in addition to its direct action on ORF3a (off-target effect), we analyzed TGF-β1 gene expression kinetics during infection. As shown in Fig. 5K, TGF-β1 expression was significantly upregulated following SARS-CoV-2 wild-type infection, consistent with previous reports linking SARS-CoV-2 to TGF-β/Smad3 signaling activation and apoptosis induction in multiple organs (57, 58). The TGF-β pathway regulates the Coordinated Lysosomal Expression and Regulation (CLEAR) network through transcription factor EB (TFEB). Analysis of CLEAR network genes from our transcriptome data revealed that SB431542 suppressed this gene network at 20 hpi, with a less pronounced effect at 40 hpi (Fig. 5L). TFEB transcript levels were significantly reduced at 12 hpi by SB431542 treatment (Fig. 5M), suggesting early inhibition of lysosomal biogenesis. The differential temporal effect of SB431542 on the CLEAR network likely reflects the ability of the compound to counteract ORF3a-mediated dysfunction during early infection, while high viral loads at later time points may partially overcome this inhibitory effect.

Since several SARS-CoV-2 proteins, including membrane protein, nucleocapsid protein, and ORF3a, are known to induce TGF-β-mediated apoptosis (59, 60), we sought to determine whether apoptosis induction facilitates viral replication. For this, we first analyzed GADD45b expression kinetics, a key positive mediator of TGF-β-induced apoptosis (13). GADD45b expression was concomitantly upregulated throughout infection but was markedly suppressed by SB431542 treatment (Fig. 5N), suggesting that SB431542 inhibits apoptosis via suppression of canonical Smad2/3 signaling and GADD45b expression.

**Fig 6:**
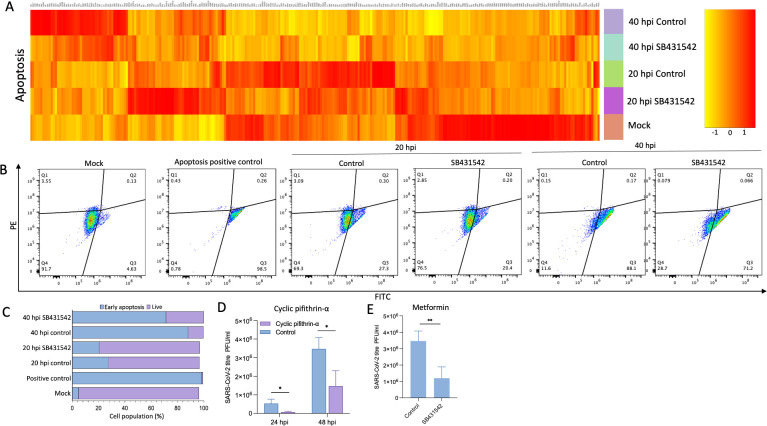
SB431542 blocks SARS-CoV-2-induced apoptosis. (**A**) RNAseq analysis: Vero cells were infected with SARS-CoV-2 wild type at 5 MOI for 1 hour, followed by treatment with DMSO or SB431542. At indicated time points, cells were scraped, and total RNA was isolated, followed by RNAseq analysis as mentioned in Materials and Methods. For analysis of apoptosis pathway genes from samples, KEGG pathway filtering was utilized. (**B**) FACS analysis of early apoptosis: Vero cells, either mock-infected or infected with SARS-CoV-2 wild type at 5 MOI, were treated with SB431542 or DMSO. At 20 and 40 hpi, cells were trypsinized and treated with JC-1 dye, followed by FACS analysis. Staurosporine treatment group was taken as a positive control. (**C**) The total cell count as a percentage is shown in a bar plot. (**D, E**) Antiviral assay: Vero cells were infected with SARS-CoV-2 wild type at 0.1 MOI for 1 hour, followed by treatment with cyclic pifithrin-α (**D**), metformin (**E**), or vehicle controls. For cyclic pifithrin-α, supernatants were harvested at 24 and 48 hpi, and for metformin, supernatants were harvested at 48 hpi, followed by virus quantification using plaque assay. Values represent means ± SD from at least three independent experiments. Statistical significance was determined using Student’s *t*-test (ns, non-significant; *P < 0.05; **P < 0.01).

To further characterize the temporal dynamics of apoptosis during infection, we examined expression of BAX, a pro-apoptotic factor regulated by GADD45b (61). Interestingly, while SB431542 suppressed BAX expression until ~24 hpi, high viral loads observed at ~36 hpi appeared to override this inhibitory effect (Fig. 5O). Given that BAX-mediated apoptosis typically requires an induction window of ~18–24 hours (62), its upregulation during mid-infection likely facilitates virus release through apoptosis during late stages. Transcriptome analysis of genes involved in the apoptosis pathway also revealed a similar pattern, demonstrating that apoptosis signaling was comparatively more active during the middle stage of infection and that SB431542 treatment suppressed it (Fig. 6A).

Next, we quantified apoptotic signatures using JC-1 dye, which measures mitochondrial membrane potential, a key indicator of early apoptosis. Flow cytometric analysis revealed that at 20 hpi, approximately 27.3% of infected cells exhibited early apoptotic signatures, which increased dramatically to 88.1% by 40 hpi (Fig. 6B). SB431542 treatment significantly reduced these apoptotic populations to 20.4% at mid-infection and 71.2% at late stages (Fig. 6C), confirming its time-dependent suppression of virus-induced apoptosis.

To establish a causal relationship between apoptosis and viral egress, we treated infected cells with cyclic pifithrin-α, a selective apoptosis inhibitor (63, 64). This treatment significantly reduced viral titers at both early (~24 hpi) and late (~48 hpi) stages of infection (Fig. 6D), phenocopying the effect of SB431542. Similarly, metformin, which induces autophagy and has been shown to normalize mitochondrial function, also suppressed viral titers at 48 hpi (Fig. 6E).

Collectively, these data demonstrate that SARS-CoV-2 exploits TGF-β-mediated apoptosis as an additional mechanism for efficient virion release during late-stage infection. SB431542 attenuates this process through canonical inhibition of TGF-β/Smad signaling, thereby suppressing the downstream apoptotic cascade. This finding bridges a critical gap between clinical observations of elevated apoptotic cell counts in lung specimens from fatal COVID-19 cases and the molecular basis of late-stage SARS-CoV-2 egress (18–20).

### SB431542 demonstrates *in vivo* efficacy against lethal IBV challenge

**Fig 7:**
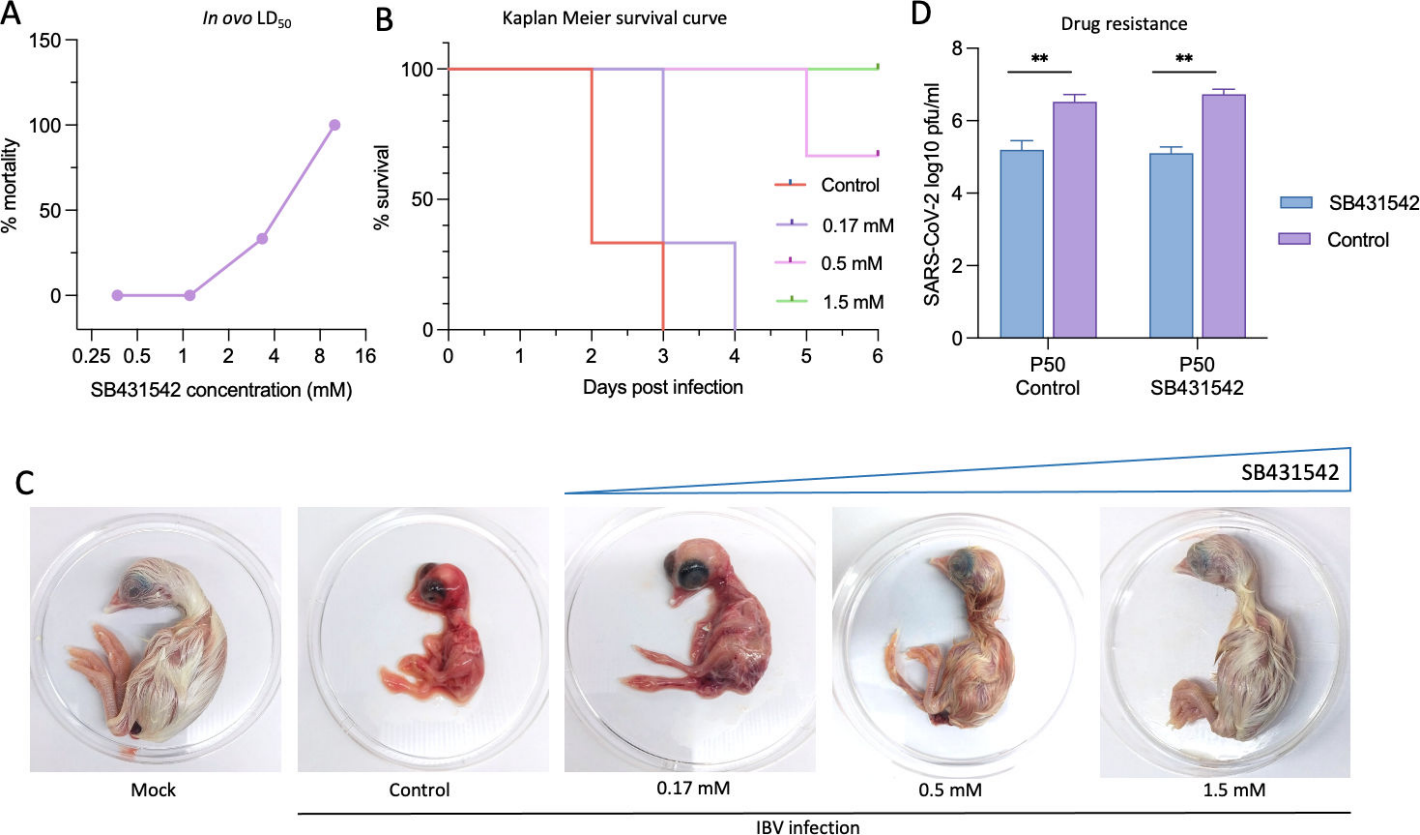
*In ovo* antiviral efficacy and drug resistance study. (**A**) Determination of LD_50_. Indicated concentrations of SB431542, in triplicate, were inoculated into 10-day-old embryonated SPF chicken eggs. The viability of the embryos was examined for up to 5 days post-inoculation, and the LD_50_ was determined to be 7.60 mM (Reed-Muench method). (**B**) Survival curve. Threefold serial dilutions of SB4531542 or DMSO were inoculated, in triplicate, in 10-days-old SPF embryonated eggs via the allantoic route, followed by infection with IBV at 100 EID_50_. The viability of the eggs was examined for 6 days post-infection. The EC_50_ was calculated to be 0.22 mM. (**C**) Morphological changes: morphological changes in the chicken embryos at different drug regimens following IBV challenge are shown. (**D**) Evaluation of SARS-CoV-2 resistance against SB431542: SARS-CoV-2 wild type was sequentially passaged 50 times in Vero cells in the presence of SB431542 or DMSO. For each passage, confluent monolayers of Vero cells were infected with SARS-CoV-2 wild type at an MOI of 0.1, followed by washing with PBS and the addition of fresh DMEM supplemented with SB431542 (0.25 uM) or 0.05% DMSO. At the end of P50, the fitness of SB431542- or DMSO-passaged viruses was evaluated again against SB431542. Values represent means ± SD from at least three independent experiments. Statistical significance was determined using Student’s *t*-test (**P < 0.01).

To assess the translational potential of SB431542 from *in vitro* to *in vivo* models, we utilized IBV, a member of the *Coronaviridae* family that causes lethal pathology in embryonated chicken eggs. Following determination of non-cytotoxic concentrations (Fig. 7A), SB431542 was administered to 10-day-old specific pathogen-free (SPF) embryonated chicken eggs via the allantoic route, concomitant with IBV challenge at 100 EID_50_ (egg infecting dose). SB431542 conferred robust, dose-dependent protection against lethal IBV infection, as evidenced by significantly improved survival rates compared to vehicle-treated controls (Fig. 7B).

Morphological assessment further corroborated these findings, revealing that SB431542 treatment dose-dependently prevented the characteristic stunted embryonic development associated with lethal IBV infection (Fig. 7C). Pharmacological evaluation demonstrated that SB431542 exhibited a lethal dose 50 (LD_50_) of 7.60 mM and an effective concentration (EC_50_) of 0.22 mM, yielding a therapeutic index (TI = LD_50_/EC_50_) of 34.54, indicating a substantial safety margin for potential therapeutic applications. These results suggest that SB431542 effectively mitigates IBV-induced lethality in embryonated chicken eggs and underscore its potential as a broad-spectrum antiviral agent against coronaviruses.

### Long-term selection pressure of SB431542 does not produce viral escape mutants

To address the critical concern of resistance emergence that plagues many direct-acting antivirals, we conducted prolonged selection pressure experiments by sequentially passaging SARS-CoV-2 wild type in Vero cells for 50 generations in the presence of either SB431542 or vehicle control (dimethyl sulfoxide [DMSO]). Remarkably, following this extensive passaging regimen, both control-passaged (P50 control) and drug-passaged (P50 SB431542) viral variants remained equally susceptible to SB431542 treatment (Fig. 7D). This exceptional genetic barrier to resistance likely stems from SB431542’s multifaceted mechanism of action, simultaneously targeting both host factors (TGF-β signaling, apoptosis) and viral component (ORF3a), thereby imposing multiple selective constraints that collectively impede the emergence of viable escape mutants. These findings starkly contrast with the rapid resistance development observed with direct-acting antivirals such as remdesivir or monoclonal antibodies, which frequently drive the evolution of escape mutants under selection pressure (65–67).

Collectively, these results highlight the potential utility of SB431542 as a robust therapeutic candidate with minimal risk of resistance development, addressing one of the most significant challenges in antiviral drug development (68, 69).

## DISCUSSION

Drug repurposing, particularly of host-directed agents (HDAs), represents a compelling antiviral strategy by establishing high genetic barriers against viral escape mutations while offering broad-spectrum activity (70–74). Unlike direct-acting antivirals, which often lead to rapid resistance development (69), numerous HDAs have been successfully repurposed as antiviral drugs or are undergoing preclinical and clinical evaluation (70, 71).

Our comprehensive investigation reveals that an established TGF-β inhibitor, SB431542 (an HDA), exhibits potent antiviral activity against coronaviruses through an unprecedented multiple-action mechanism: inhibition of viral entry via canonical TGF-β/Smad signaling modulation, direct binding to viral ORF3a protein, which disrupts lysosomal function, and attenuation of TGF-β-induced apoptosis that facilitates late-stage viral release. Besides Vero cells used in the present study, the antiviral efficacy of SB431542 was shown in Caco-2, Calu-3, and Huh-7 cells (8), supporting its therapeutic potential, though mechanisms remained unclear.

Our time-of-addition and step-specific assay revealed that SB431542 suppressed maximum SARS-CoV-2 replication when administered either before infection or within early post-infection periods. While its inhibitory effect on viral entry through TGF-β/Smad-mediated modulation of furin expression was reported previously (8), our study uncovered two additional mechanisms targeting the later stages of the viral life cycle. Notably, despite comparable viral protein synthesis, we observed a significant reduction in both intracellular and extracellular virions following SB431542 treatment. This indicates potential interference with viral assembly and egress pathways rather than replication machinery.

The observed disruption of nucleocapsid-RNA association in RNA-IP assays provided the first mechanistic insight into how SB431542 impairs virion assembly (Fig. 4C). Given the established role of ORF3a in lysosomal dysfunction and autophagy subversion during SARS-CoV-2 infection, we hypothesized that SB431542 might directly target this viral protein. Our *in silico* analyses revealed that SB431542 binds to ORF3a with high affinity (ΔG = −13.32 kcal/mol), outcompeting bacteriochlorin (ΔG = −5.28 kcal/mol) at a site overlapping with the Saposin-A interaction interface (40). Experimental validation through ITC assay further confirmed this computational prediction, revealing direct SB431542-ORF3a binding with a K_D_ of 220 ± 68.1 nM, correlating with the observed antiviral efficacy. This binding site is critically positioned to disrupt the canonical role of ORF3a in inhibiting autophagosome-lysosome fusion and promoting lysosomal deacidification, the processes essential for preserving virion integrity during egress (40). While our study specifically investigated the inhibitory action of SB431542 on ORF3a, a previous study demonstrated that the overexpression of SARS-CoV-2 ORF3a significantly increased viral titers (75), supporting its functional importance in the viral life cycle. These evidences collectively reinforce our mechanistic model positioning ORF3a as a pivotal viral factor and promising target for antiviral intervention.

Immunofluorescence studies also validated this mechanism, demonstrating that SB431542 treatment normalized LC3-GFP distribution patterns, restored perinuclear LAMP-1 localization, and reacidified lysosomes in infected cells (Fig. 2). These effects collectively reversed the lysosomal dysfunction induced by SARS-CoV-2 infection, consistent with the predicted interaction of the compound with ORF3a. The relevance of this pathway was further confirmed when metformin, a known autophagy inducer, similarly suppressed viral titers (Fig. 5D) (50). These findings complement recent studies identifying lysosomal exocytosis as a distinctive egress mechanism for β-coronaviruses and suggest that therapeutic modulation of this pathway represents a viable antiviral strategy (3).

A particularly novel aspect of our study is the characterization of temporal dynamics in SARS-CoV-2 egress mechanisms. While previous investigations have primarily focused on early infection stages (≤24 hpi) (2, 3, 54), our one-step growth curve analysis revealed a substantial 340% increase in viral titers between 36 and 48 hpi. Coupled with the emergence of cytopathic effects only after ~42 hpi, this temporal progression suggested potentially additional viral egress strategies during prolonged infection. Indeed, our JC-1 staining experiments demonstrated progressive induction of apoptosis, with early apoptotic signatures increasing from 27.3% at 20 hpi to 88.1% by 40 hpi. Mechanistically, we observed time-dependent upregulation of TGF-β1 and its downstream effector GADD45b during infection, with subsequent induction of the pro-apoptotic factor BAX (Fig. 5I, J, and 6A) (61).

The relationship between apoptosis and late-stage viral release was confirmed when cyclic pifithrin-α, a selective apoptosis inhibitor (64), significantly reduced viral titers (Fig. 6D). SB431542 treatment similarly suppressed apoptotic signatures and reduced viral titers, indicating that inhibition of TGF-β-mediated apoptosis constitutes a third mechanism through which this compound exerts antiviral effects. This finding bridges an important gap between the clinical observation of elevated apoptotic cell counts in lung specimens from fatal COVID-19 cases (16, 18–20) and the molecular basis of late-stage SARS-CoV-2 egress, suggesting that increasing viral load-induced apoptosis serves as a release mechanism complementary to lysosomal exocytosis during sustained infection.

Furthermore, the translational potential of SB431542 was validated in an IBV infection model using embryonated chicken eggs, where dose-dependent protection against lethal viral challenge was observed (Fig. 7C). The calculated therapeutic index of 34.54 highlights a favorable safety margin, although further studies in mammalian models will be necessary to fully evaluate its pharmacological profile. Intriguingly, the efficacy of SB431542 has been previously demonstrated in mice against SARS-CoV-2 spike-mediated barrier dysfunction and vascular leak, where it was shown to reduce spike protein-triggered endothelial hyperpermeability and endothelial glycocalyx layer disruption (76). These evidences, combined with our *in vivo* studies, collectively suggest potential pharmacological intervention of SB431542 to alleviate SARS-CoV-2 pathology. Perhaps most significantly, sequential passaging of SARS-CoV-2 for 50 generations under SB431542 selection pressure failed to generate resistant variants (Fig. 7D). This high genetic barrier to resistance likely stems from SB431542’s multifaceted mechanism of action targeting both host factors (TGF-β signaling, apoptotic machinery) and viral component (ORF3a). This contrasts with direct-acting antivirals such as remdesivir or monoclonal antibodies, which frequently drive the evolution of escape mutants under selection pressure (65–67).

Collectively, our findings establish SB431542 as a promising antiviral candidate with a remarkable EC_50_ of 751.8 nM against SARS-CoV-2 wild type, significantly more potent than the FDA-approved remdesivir (EC_50_: 1.65 µM) in comparable Vero cell models (28). The compound’s unique triple-mechanism approach—inhibiting viral entry via TGF-β/Smad signaling modulation, disrupting ORF3a-mediated lysosomal dysfunction affecting assembly and egress, and attenuating TGF-β-induced apoptosis during late-stage infection—provides multiple barriers against viral replication while minimizing resistance development.

However, certain limitations warrant further investigation. While our study demonstrates efficacy *in vitro* and *in vivo* using embryonated chicken eggs as a model system, additional preclinical studies using mammalian models are necessary to evaluate pharmacokinetics, toxicity profiles, and efficacy against emerging SARS-CoV-2 variants. Additionally, comprehensive toxicological studies will be needed to assess potential side effects associated with systemic TGF-β inhibition, particularly in the context of prolonged treatment.

## MATERIALS AND METHODS

### Cells and viruses

Vero cells (African green monkey kidney cells) were cultured in Dulbecco’s modified Eagle medium (DMEM) supplemented with 10% fetal bovine serum (FBS) and antibiotics. The SARS-CoV-2 wild type (2020) and Omicron BA.1 (2022) variants, previously isolated from clinical samples by our group and accessioned as VTCCAVA318 and VTCCAVA336, respectively, at the National Center for Veterinary Type Cultures (NCVTC), Hisar, India (https://ncvtc.org.in/), were available for use in this study. The viruses were propagated in Vero cells under Biosafety Level 3 (BSL-3) conditions at the National Center for Veterinary Type Cultures (NCVTC), ICAR-National Research Center on Equines, Hisar, India. Viral titers were quantified as plaque-forming units per milliliter by plaque assay.

For *in vivo* studies, IBV, a member of the *Coronaviridae* family, was used to infect SPF embryonated chicken eggs. IBV was propagated and quantified as the egg infectious dose 50 (EID_50_) per mL.

### Inhibitors

SB431542 was purchased from Sigma-Aldrich and dissolved in DMSO. Bafilomycin, cyclic pifithrin-α, metformin, and Staurosporine were procured from MedChemExpress.

### Cytotoxicity assay

The cytotoxicity of drugs in Vero cells was determined using the MTT assay. Briefly, Vero cells were seeded into 96-well plates and treated with serial dilutions of inhibitors for 96 hours. Cell viability was measured by adding MTT reagent (5 mg/mL) to each well, followed by incubation at 37°C for 4 hours. The formazan crystals formed were dissolved in DMSO, and absorbance was measured at 570 nm using a microplate reader. The half-maximal cytotoxic concentration (CC_50_) was calculated using the Reed-Muench method.

### Plaque assay

Plaque assays were performed to quantify SARS-CoV-2 titers. Confluent monolayers of Vero cells in 12-well plates were infected with serial dilutions of virus-containing supernatants for 1 hour at 37°C. After infection, supernatants containing viruses were removed, and cells were overlaid with L-15 media containing 2% agarose and incubated for 96 hours at 37°C. Plaques were visualized by staining with crystal violet solution and counted to determine viral titers.

### Time-of-addition assay

Confluent monolayers of Vero cells were infected with SARS-CoV-2 at an MOI of 5, followed by washing with phosphate-buffered saline (PBS) five times, supplemented with fresh DMEM, and treated with SB431542 or vehicle control (DMSO) at various time points post-infection (−0.5 h to 36 hpi). Supernatants were collected at 48 hpi, and viral titers were quantified by plaque assay.

### Virus step-specific assays

For attachment assay, Vero cell monolayers were pre-treated with SB431542 or vehicle control for 1 hour, followed by SARS-CoV-2 infection (MOI 5) at 4°C for 1 hour. After five PBS washes to remove unbound virus, cell lysates were prepared by freeze-thaw cycling, and viral titers were quantified by plaque assay.

For virus entry studies, prechilled Vero cell monolayers were infected with SARS-CoV-2 (MOI 5) at 4°C for 1 hour to permit attachment. Following five PBS washes, cells were incubated with SB431542 or vehicle control at 37°C for 1 hour to allow virus entry. After removing extracellular virus by PBS washing, cells were maintained in inhibitor-free DMEM. Infectious virus particles in supernatants were quantified by plaque assay at 12 hpi.

To assess virus release, SARS-CoV-2-infected Vero cells (MOI 5) were incubated until 8 hpi. Cells were then washed and treated with SB431542 or vehicle control. Supernatants were collected at 4 hours post-treatment for plaque assay quantification.

For viral protein synthesis analysis, SARS-CoV-2-infected (MOI 5) or mock-infected Vero cells were treated with SB431542 or vehicle control at 3 hpi. Cell lysates were prepared at 12 hpi for western blot analysis of viral and cellular proteins.

To quantify viral RNA/DNA, SARS-CoV-2-infected Vero cells (MOI 5) were treated with SB431542 or vehicle control at 2 hpi. At 12 hpi, total RNA/DNA was extracted. cDNA was synthesized using oligo dT primers (Fermentas, Hanover, USA) for mRNA quantification. SARS-CoV-2 *N* gene and β-actin expression were quantified by quantitative real-time PCR (qRT-PCR) as described previously (77).

### Lysosomal function assays

To assess lysosomal function, Lysotracker Red DND-99 dye (Thermo Fisher Scientific) and Hoechst dye were used to stain acidic lysosomes and nuclei, respectively, in infected Vero cells treated with SB431542 or vehicle control. Cells were incubated with the dye for 30 minutes at 37°C according to the manufacturer’s protocol, washed with PBS, and visualized under a fluorescence microscope.

For transcriptomic analysis of autophagy-related genes, RNA was extracted from infected cells using TRIzol reagent (Thermo Fisher Scientific). cDNA synthesis was performed using oligo-dT primers, followed by qRT-PCR using SYBR Green Master Mix (Bio-Rad). Gene expression levels were normalized to β-actin as a housekeeping gene.

### Apoptosis assays

Mitochondrial membrane potential was assessed using JC-1 dye (Thermo Fisher Scientific). Infected Vero cells treated with SB431542 or vehicle control were harvested at mid-infection (20 hpi) and late stages (40 hpi). Cells were stained with JC-1 dye according to the manufacturer’s protocol and analyzed using a BD FACSARIA flow cytometer. The percentage of early apoptotic cells was determined based on red-to-green fluorescence ratios. Staurosporine-treated cells were taken as a positive control.

### RNA immunoprecipitation assay

RNA-IP assays were performed to evaluate the interaction between viral RNA and nucleocapsid protein. Infected Vero cells treated with SB431542 or vehicle control were crosslinked with formaldehyde to stabilize RNA-protein interactions at 12 hpi by incubating with 1% formaldehyde for 10 minutes and quenched with 125 mM glycine. Cells were lysed in immunoprecipitation/lysis buffer (150 mM NaCl, 50 mM Tris-HCl [pH 7.5], 5 mM EDTA, 0.5% NP-40, 1% Triton X-100, and protease/phosphatase inhibitor cocktail) by incubating for 30 minutes followed by sonication (Qsonica Q500) using six 15 second pulses at 40% amplitude, clarified by centrifugation (12,000 × *g*, 10 minutes), and supplemented with 10 units of RiboLock RNase Inhibitor (Invitrogen, Carlsbad, USA). Cell lysates were immunoprecipitated using SARS-CoV-2 anti-nucleocapsid antibody conjugated to Protein A Sepharose beads (Sigma-Aldrich). α-MNK1 antibody and IP buffer served as non-reactive and bead controls, respectively. Following five washes with IP buffer, RNA-protein complexes were reverse crosslinked, and RNA was extracted using TRIzol reagent for downstream qRT-PCR analysis. Values were normalized to input RNA. All experiments were performed in triplicate.

### Cloning and overexpression of ORF3a

SARS-CoV-2 wild-type ORF3a was cloned into the pDONR221 vector and subsequently transferred to the pDEST40 vector using GATEWAY cloning technology (Invitrogen, Carlsbad, CA, USA) according to the manufacturer’s instructions. The ORF3a was amplified with attB-flanked primers (forward primer 5′-GGGGACAAGTTTGTACAAAAAAGCAGGCTTCACCATGGATTTGTTTATGAGAATCTTCACAATTGG-3′ and reverse primer 5′-GGGGACCACTTTGTACAAGAAAGCTGGGTGCAAAGGCACGCTAGTAGTCGTC-3′). Cloned constructs were verified by Sanger sequencing.

### Immunofluorescence studies

HeLa cells grown in 4-chambered slides at 5% confluency in DMEM supplemented with FBS were transfected with 1 µg of LC3-GFP (addgene #11546) and pDEST40:ORF3a or empty plasmid using Lipofectamine 3000. At 24 hours post-transfection (hpt), cells were treated with SB431542 or vehicle control, and at 48 hpt, cells were subjected to immunofluorescence studies for visualization of LC3-GFP puncta. In case of transfection in Vero cells with LC3-GFP, at 48 hpt, SARS-CoV-2 wild-type infection was given at 5 MOI in the presence of SB431542 or vehicle control, and at 12 hpi, cells were subjected to immunofluorescence studies as described previously (78). For probing vgRNA and N gene, SARS-CoV-2 wild-type infected Vero cells treated with SB431542 or vehicle control were fixed and permeabilized at 12 hpi and probed using J2 antibody and anti-nucleocapsid antibody. Nuclei were counterstained with DAPI.

### *In vivo* antiviral efficacy in embryonated chicken eggs

SPF embryonated chicken eggs (10 days old) were procured from Indovax Pvt. Ltd., Hisar, India. To determine the lethal dose 50 (LD_50_) of SB431542, eggs were inoculated via the allantoic route with serial dilutions of SB431542 or DMSO as a vehicle control. Egg viability was monitored daily for up to 6 days post-inoculation.

For antiviral efficacy studies, eggs were infected with IBV at an EID of 100 via the allantoic route and treated with SB431542 or DMSO immediately post-infection. Eggs were monitored for survival and fetal growth for up to 6 days post-infection.

### RNA extraction, library preparation, and Illumina sequencing

Vero cells (80%–85% confluent) were infected with SARS-CoV-2 wild type (MOI 5), treated with SB431542 at 1 hpi, and harvested at 20 and 40 hpi for total RNA extraction using TRIzol reagent (Takara, China).

Poly(A) mRNA was enriched from total RNA (500 ng) using the NEBNext Poly(A) mRNA Magnetic Isolation Module (New England Biolabs, MA, USA) according to the manufacturer’s protocol. Libraries were prepared using the NEBNext Ultra II RNA Library Prep Kit. Enriched mRNA was fragmented in magnesium-based buffer at 94°C for 10 minutes using NEBNext Random Primers to generate ~300 nucleotide inserts. The fragmented RNA was reverse transcribed into first-strand cDNA and was followed by double-stranded DNA conversion and purification using 1.8X AMPure XP beads (Beckman Coulter, CA, USA). The purified dsDNA was subjected to end repair, 3′ adenylation, and loop adapter ligation.

Following USER enzyme treatment, adapter-ligated products were size-selected using AMPure XP beads to obtain 400–600 bp libraries. Libraries were amplified through 12 PCR cycles using NEBNext Ultra II Q5 Master Mix and Multiplex Oligos, then purified with 0.9× AMPure XP beads and eluted in 15 µL of 0.1× TE buffer.

Adapter ligated products were treated with USER enzyme and size-selected using AMPure XP beads (Beckman Coulter, CA, USA) to target a library size of 400–600 bp. The cDNA libraries were amplified through 12 PCR cycles using NEBNext Ultra II Q5 Master Mix and NEBNext Multiplex Oligos for Illumina. The amplified libraries were purified with 0.9× AMPure XP beads and eluted in 15 µL of 0.1× TE buffer.

Library quality was assessed using a Qubit Fluorometer (Invitrogen, Life Technologies, USA) for quantification and a Tapestation system (Agilent Technologies, USA) with an HSDNA kit for size distribution analysis. Pooled libraries underwent cluster generation on the c-Bot system, followed by paired-end sequencing (2 × 150 bp) on an Illumina HiSeq X10. Demultiplexing and adapter trimming were performed using CASAVA v1.8.2 (Illumina Inc.).

### RNA-seq data analysis

Raw read quality was assessed using FastQC (v0.11.8) to examine base quality score distribution, sequence quality score distribution, average base content per read, and GC content distribution. Adapter sequences (AGATCGGAAGAGC) were removed using Trim Galore (v0.6.2), which also performed automated quality trimming. Reads shorter than 20 bp or with low-quality ends (Phred score <20) were discarded. The *Chlorocebus sabaeus* reference genome (Ensembl release 110) and SARS-CoV-2 wild-type genome were indexed using BWA (v0.7.17), and pre-processed reads were aligned using BWA-MEM with default parameters. Mapped reads were quantified at the gene level using Samtools (v0.1.19).

Differential expression analysis was performed using DESeq (v1) to identify genes with significant expression changes. Differentially expressed genes (DEGs) were defined using threshold criteria of fold change ≥2 and *P* value <0.05. DEGs were functionally annotated using BLASTx (v2.2.29+) against the NR database, with annotations retrieved from UniProt and Kyoto Encyclopedia of Genes and Genomes (KEGG) databases. Gene Ontology (GO) terms were visualized using WEGO with a log10-scaled y-axis. Expression patterns were visualized through volcano plots and MA plots generated using custom R scripts, while heatmaps were created using MeV software (v4.8.1).

### Validation of RNA-seq data

To validate RNA-seq data, four genes were randomly chosen from each CLEAR network, apoptosis, and autophagy pathways based on heat map analysis. Gene variants were aligned in Geneious Prime software, and primers were designed based on conserved regions. qRT-PCR was performed after harvesting SARS-CoV-2 wild-type infected cells at indicated time points with or without drug treatment and normalized to the β-actin gene (housekeeping control). The primer sequences are provided in Table S1.

### Generation of protein-ligand complexes

To investigate the binding interactions of SB431542 with SARS-CoV-2 wild-type ORF3a, protein-ligand complexes were generated using the diffusion-based structure prediction tool Boltz (35). This approach leverages machine learning models trained on data sets of bound complexes by simulating “diffusion” from a disordered state to a bound state. The protein template was based on the experimentally determined cryo-EM structure of ORF3a dimer (PDB ID: 6XDC, resolution 2.9 Å) (79). To maintain the physiologically relevant dimeric state of ORF3a, structure prediction was performed with two copies of both the protein sequence and ligand SMILES string. For analysis of single ligand binding (SB431542), the second ligand was subsequently removed from the complex. A total of 25 structural models were generated, and as a reference compound, we modeled bacteriochlorin binding to ORF3a using identical protocols, as bacteriochlorin is structurally similar to chlorin compounds previously reported to interact with the chloride binding site of ORF3a (34, 80).

The Boltz-generated complexes were further refined to ensure compatibility with downstream molecular dynamics simulations. This approach was chosen over traditional docking methods (e.g., GLIDE, AutoDock, and RosettaDock) due to their limitations in accurately predicting binding affinities when experimental data is unavailable. Diffusion-based modeling provides an alternative capable of generating high-confidence protein-ligand complexes with performance comparable to AlphaFold3 (81).

### Molecular dynamics simulation protocol

MD simulations were performed using GROMACS with the CHARMM36 force field for proteins (82). For small molecule parameterization, we employed espaloma, a machine learning-based approach that generates classical force field parameters (83). The all-atom simulations followed an adjusted protocol based on established methodologies (84, 85).

The parameters can be found in the GitHub repository https://github.com/carlocamilloni/labtools/tree/main/mdps/atomistic. The protocol began with a series of minimization steps in vacuum, consisting of steepest descent minimization, followed by conjugate gradient minimization (repeated twice), and a single minimization using the LBFGS method, all implemented within GROMACS. After these initial minimization steps, the protein-ligand complex was solvated and neutralized with ions, and the same series of minimization procedures was repeated for the solvated system.

System equilibration was conducted in two phases: an initial 2 ns simulation followed by a 0.5 ns relaxation simulation, both performed in the NPT ensemble using scripts 3-pr-npt.mdp and 4-npt-relax.mdp, respectively. Production simulations were then carried out for 500 ns in the NPT ensemble. All simulations were performed at a temperature of 300 K and a pressure of 1 bar.

For trajectory analysis, we calculated several standard metrics, including RMSD to assess overall structural stability, Rg to detect potential protein unfolding, protein-ligand contacts (using a 4 Å distance cut-off), and RMSF to evaluate residue mobility. All analyses were performed using custom Python scripts with the biotite library. Simulation protocols and scripts are available at the GitHub repository https://github.com/entropybit/md_sarscov2_orf3a.

### Binding free energy calculations

Multiple computational approaches were employed to estimate binding affinities. First, MM-GBSA calculations were performed on the last 1,000 frames of each simulation using gmx_MMPBSA (37, 38). The implicit solvent model was employed with a salt concentration of 0.15 M. Next, non-equilibrium pulling simulations were conducted by applying a harmonic restraint between the center of mass of the ligand and a reference point outside the binding pocket (86). The restraint was moved at a constant velocity of 10 nm/ns over 2 ns with a force constant of 1,000 kJ/mol/nm². Only a single pulling simulation was performed as a preparation step for the subsequent umbrella run. And finally, for more accurate binding free energy estimation, we used umbrella sampling on frames selected from the pulling trajectory (39). Initially, a total of 20 windows with equidistant spacing over the generated pulling trajectory were chosen. After evaluation of the resulting histograms, windows were added manually at frames that best represent the center of gaps between histograms. A total of 45 windows for SB431542 and 42 windows for Bacteriochlorin were simulated using this approach. For an exact documentation as well as a result of the performed umbrella sampling runs, see the GitHub repository https://github.com/carlocamilloni/labtools/tree/main/mdps/atomistic. Each window was sampled for 10 ns with a restraint force constant of 5 kcal/mol/Å². The weighted histogram analysis method was used to reconstruct the PMF and calculate binding free energies (ΔG). The binding free energy was estimated by calculating the difference between PMF values of bound and unbound states, providing a more thermodynamically rigorous assessment of ligand affinity.

All trajectory analyses, including RMSD, RMSF, Rg, and protein-ligand contacts, were performed using customized Python scripts utilizing the Biotite library.

### Isothermal titration calorimetry assay

The direct binding interaction between the SARS-CoV-2 ORF3a protein and SB431542 was characterized using isothermal titration calorimetry. Recombinant SARS-CoV-2 ORF3a protein (full-length, residues 1–275, accession no. QHD43417.1) was obtained from MRC PPU Reagents and Services (Dundee, Scotland, UK). To ensure accurate thermodynamic measurements and minimize systematic artifacts from buffer mismatch, both ORF3a protein and SB431542 ligand were dialyzed extensively and prepared in identical buffer compositions (50 mM HEPES, 150 mM NaCl, pH 7.4, 0.025% DDM, 0.3% DMSO). Final concentrations were 3 µM for ORF3a protein (sample cell) and 30 µM for SB431542 ligand (injection syringe).

ITC measurements were performed using a Microcal PEAQ-ITC microcalorimeter (Malvern Panalytical, UK) at 25°C with a reference power of 10 μcal/second. Prior to experiments, all solutions were degassed under vacuum with gentle stirring for 15 minutes to prevent bubble formation during titration. For protein-ligand binding experiments, the 30 µM concentration of SB431542 ligand (in syringe) was titrated against 3 µM of ORFA3a protein (in cell). After thermal equilibration, a total of 13 injections of syringe samples were titrated in the cell, with one injection of 0.4 µL followed by 12 subsequent injections of 3 µL. Each injection was spaced 150 seconds apart with continuous stirring at 600 rpm. Control experiments were performed by titrating SB431542 into buffer alone under identical conditions to determine the heat of dilution, which was subsequently subtracted from protein-ligand binding isotherms. Raw thermograms were processed and analyzed using MicroCal PEAQ-ITC analysis software (version 1.41, Malvern Panalytical). Binding parameters, including stoichiometry (n), binding affinity (Ka), enthalpy (ΔH), and entropy (ΔS), were determined by fitting the integrated heat data to a one-site binding model.

### Statistical analysis

All experiments were conducted in triplicate unless otherwise specified. Data are presented as mean ± standard deviation (SD). Statistical significance between groups was determined using Student’s *t*-test (*P* < 0.05 considered significant).

## Data Availability

All data supporting the findings of this study are available within the paper and from the corresponding author upon request. There are no restrictions on obtaining access to the primary data.

## SUPPLEMENTAL MATERIAL

Figures S1 to S3 jvi.00529-25-s0001.pdf Fig. S1: SB431542 targets SARS-CoV-2 ORF3a. Fig. S2: SB431542 suppresses perinuclear localization of vgRNA. Fig. S3: Quantification of genes related to CLEAR network, apoptosis, and autophagy pathways.

Table S1 jvi.00529-25-s0002.docx Primer sequences of genes related to CLEAR network, apoptosis, and autophagy pathways for RNA-seq data validation.

ASM does not own the copyrights to Supplemental Material that may be linked to, or accessed through, an article. The authors have granted ASM a non-exclusive, world-wide license to publish the Supplemental Material files. Please contact the corresponding author directly for reuse.
