# Supplementary material for: TGF-β inhibitor SB431542 suppresses SARS-CoV-2 replication through multistep inhibition
Source: J Virol. 2025 Aug 29;99(9):e00529-25. doi: 10.1128/jvi.00529-25 (PMC12455996; doi:10.1128/jvi.00529-25)
Supplement: Figures S1 to S3 — Fig. S1: SB431542 targets SARS-CoV-2 ORF3a. Fig. S2: SB431542 suppresses perinuclear localization of vgRNA. Fig. S3: Quantification of genes related to CLEAR network, apoptosis, and autophagy pathways. [file jvi.00529-25-s0001.pdf]

Supplementary Figure 1

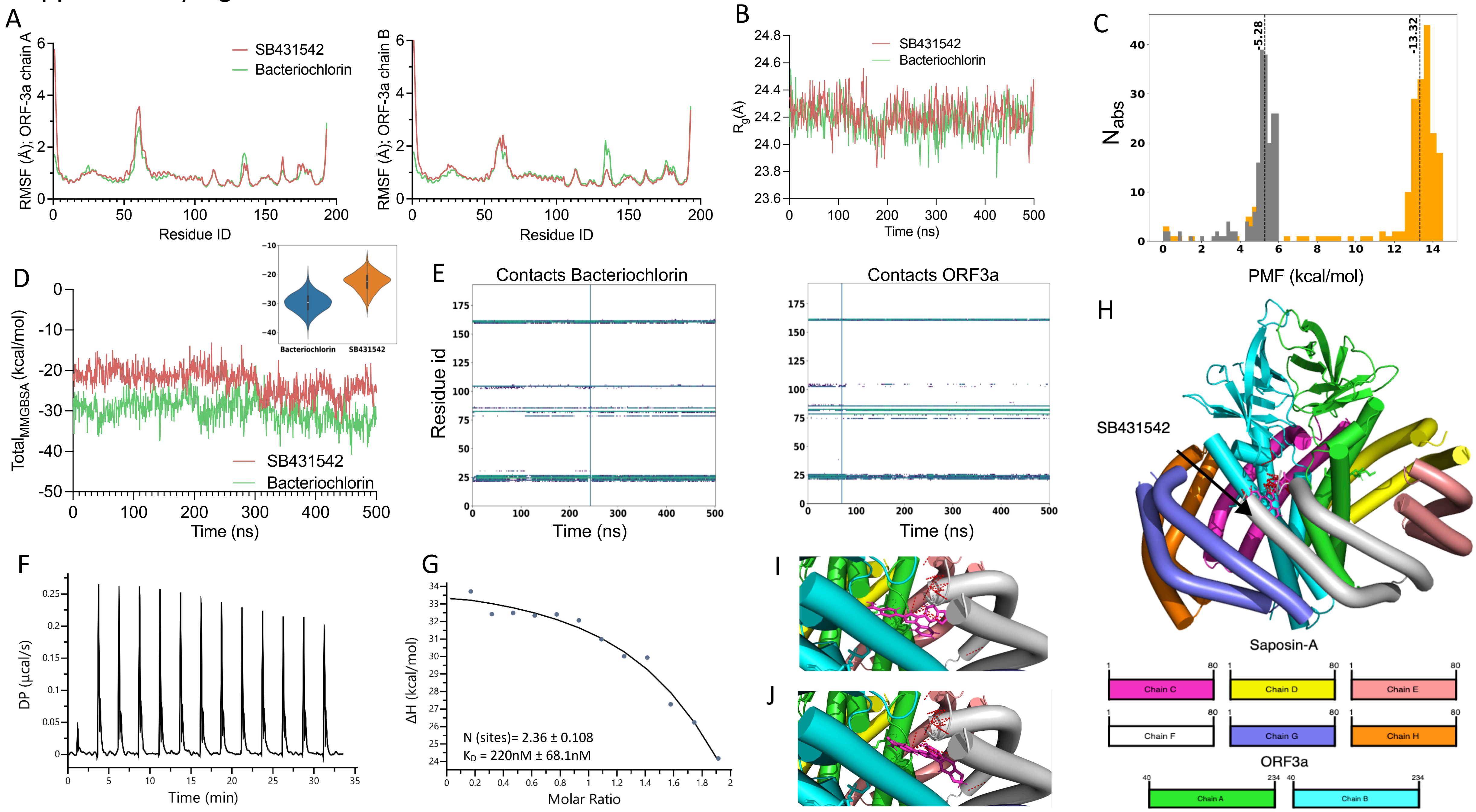

Supplementary figure 1 | SB431542 targets SARS-CoV-2 ORF3a.

A. Root mean square fluctuation (RMSF): The root mean square fluctuation for both ORF3a chains A and B is illustrated for both performed 500 ns MD simulations. Albeit showing deviations the location of the peaks is the same for all shown RMSF profiles, which indicates only minimal deviation between the ORF3a complexes observed in both trajectories. **B. Radius of gyration (R_g):** Vthe radius of gyration of the ORF3a protein complex is plotted for both performed simulation. This shows an oscillation around a flat line indicating no change in conformation of the protein complex. **C ΔG histograms:** A histogram of all possible estimates of ΔG from the potential of mean forces profile is illustrated. These estimates are gained by calculating all differences to the first PMF value. The dotted lines indicate the average value chosen as estimator for the actual ΔG value gained from the umbrella sampling run. **D. MM/GBSA interaction energy:** The total interaction energy between protein and ligand over time is illustrated gained from the performed MM/GBSA runs. **E. Protein-ligand interaction profiles:** Both figures show the interaction profiles of SB431542 and Bacteriochlorin over the 500 ns of simulated time. On the y axis the ORF3a residue id is indicated and on the x-Axis the time in nano seconds. A line color coded by number of contacts between ligand and the indicated ORF3a residue is illustrated over time in this plot. The vertical line indicates the time of maximum overall number of contacts between protein and ligand. **F, G. Calorimetric titration isotherms of binding of SARS-CoV-2 ORF3a and SB431542. F.** Thermogram of binding of SB431542 to ORFA3 protein representing heat changes during injections. **G.** Isotherm derived from integrated thermogram data. Plot represents binding enthalpy against to molar ratio of ligand to protein. The concentration of protein ORFA3a is 3μM and the concentration of ligand is 30 μM. **H. SB431542 complex aligned with ORF3a – Saposin-A complex:** This illustration shows an alignment of the generated ORF3a – SB431542 complex with the resolved ORF3a – Saposin-A complex based on structure 8EQU from the pdb. Only the proteins from 8EQU are shown and the according chains are illustrated by color and residues in the blocks above the illustration. The ligand is colored in magenta and indicated by the arrow. **I, J SB431542 located in between ORF3a – Saposin-A interface at ininitial and end state of MD simulation:** Both pictures show a zoom of the ORF3a – Saposin-A protein-protein interacation interface. The protein interactions are visualized by red dashed lines and it is clearly visible that in both visualized states at the starting point of the simulation (t=0 ns) and the final state of the simulation (t=500 ns) the SB431542 compound overlaps with the interactions visible in the resolved structure 8EQU.

Supplementary Figure 2

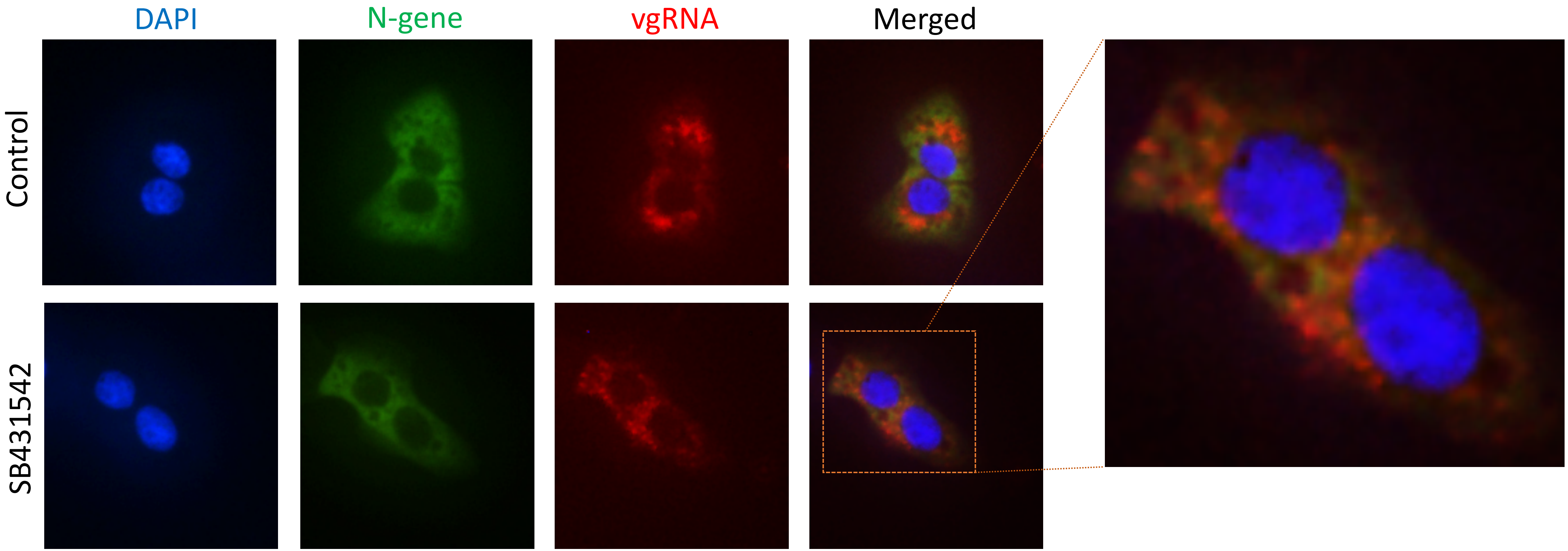

Supplementary figure 2 | SB431542 suppress perinuclear localization of vgRNA.

Immunofluorescence imaging: Distribution of SARS-CoV-2 N gene (green) and vgRNA (red) in infected Vero cells at 12 hpi. Vero cells were inoculated with 5 MOI of SARS-CoV-2 wild-type and incubated at 37°C. At 1 hpi cells were extensively washed with PBS and further incubated for 11 hours after supplementing with fresh DMEM (control, upper panel) and SB431542 (lower panel). At 12 hpi, cells were fixed and permeabilized followed by probing N proteins with anti-N antibody and vgRNA with J2 antibody. Nuclei were counterstained with DAPI (blue).

Supplementary Figure 3

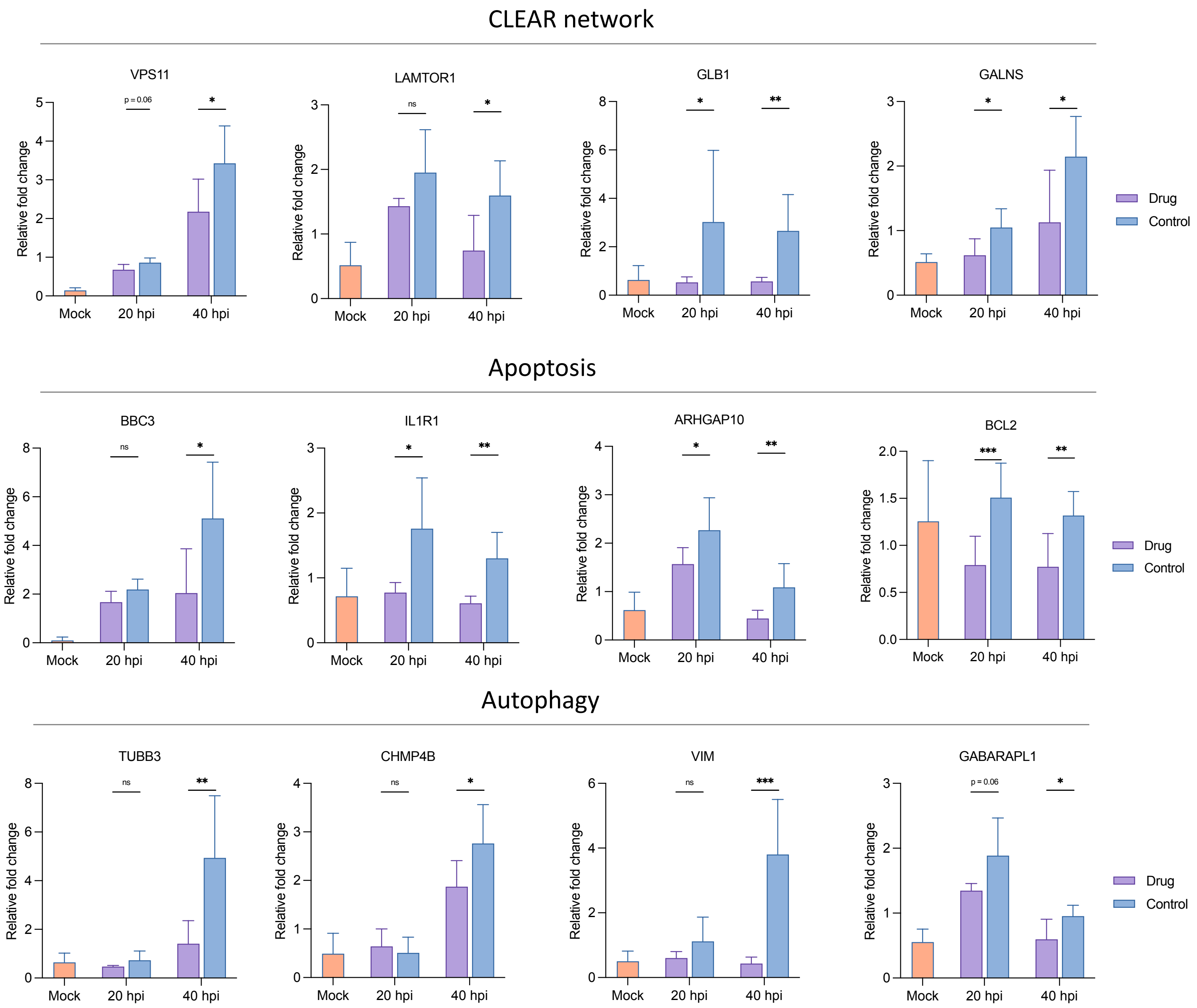

Supplementary figure 3 | Quantification of genes related to CLEAR network, apoptosis and autophagy pathways

Vero cells were infected with SARS-CoV-2 wild-type at 1 MOI for 1 hour followed by treatment with DMSO or SB431542. At indicated time-points, cells were scraped and RNA was isolated, followed by cDNA library preparation using oligo(dT). Genes related to CLEAR network, apoptosis and autophagy pathways were amplified from the harvested cells and normalized to β-actin gene (housekeeping control). Relative fold-change was calculated using the ΔΔCt method. Values represent means ± SD from at least three independent experiments. Statistical significance was determined using Student's t-test (ns = non-significant, $^*P < 0.05$; $^{**}P < 0.01$; $^{***}P < 0.001$).
