# Supplementary material for: TGF-β inhibitor SB431542 suppresses SARS-CoV-2 replication through multistep inhibition
Source: J Virol. 2025 Aug 29;99(9):e00529-25. doi: 10.1128/jvi.00529-25 (PMC12455996; doi:10.1128/jvi.00529-25)
Supplement: Table S1 — Primer sequences of genes related to CLEAR network, apoptosis, and autophagy pathways for RNA-seq data validation. [file jvi.00529-25-s0002.docx]

| **Gene** | **Forward Primer (5’-3’)** | **Reverse Primer (5’-3’)** |
| --- | --- | --- |
| VPS11 | ACGTGATCCGCAAGTTTCTAGATG | TGAGGAGCAGGGTGGTATGG |
| LAMTOR1 | AGAGGAGCGGAAGCTGCTG | AGGCTGCTGCTCAGCACAG |
| GLB1 | TCATCTCTCCAAACCTGTTGAGC | TCAGGATGTGCTGGGGCAC |
| GALNS | AGCCAACCTCACCCAGATCTAC | TCCCTCATCCCTCCTTCAAACG |
| BBC3 | TCAGCCCTCGCTTTCGCTG | TCTTGTCTCCGCCGCTCG |
| IL1R1 | TCCTCTTAACCCAAATGAATACAAAGGC | TGGCTTCTGCATTATAACACAAGTTAGG |
| ARHGAP10 | AGAGGCATACTGACTCCATTGACAG | TTCCTTCTGGTCTTGGGATGATGG |
| BCL2 | AGTGATGAAGTACATCCACTATAAGCTG | TCTCCACACACATGACCCCAC |
| TUBB3 | TGCGGAGCTAGTGGACTCG | AGCGTGGCATTGTAGGGCTC |
| CHMP4B | AGCAAGAAACAGGAGTTCCTAGAG | ACCTCGGTGTTGGTGTTGGC |
| VIM | ATTGCAGGAGGAGATGCTTC | TCAAAAAGGCAATCTCTTCTTGC |
| GABARAPL1 | TTGTAGAGAAGGCTCCAAAAGCC | TCATACAGTTGGCCCATGGTAG |

**Supplementary Table 1 :** Primer sequences of genes related to CLEAR network, apoptosis and autophagy pathways for RNA-seq data validation.
